# Neutrophil extracellular traps aggravate intestinal epithelial necroptosis in ischaemia–reperfusion by regulating TLR4/RIPK3/FUNDC1‐required mitophagy

**DOI:** 10.1111/cpr.13538

**Published:** 2023-09-10

**Authors:** Chengnan Chu, Xinyu Wang, Fang Chen, Chao Yang, Lin Shi, Weiqi Xu, Kai Wang, Baochen Liu, Chenyang Wang, Dongping Sun, Jieshou Li, Weiwei Ding

**Affiliations:** ^1^ Division of Trauma and Acute Care Surgery, Department of Surgery, Jinling Hospital, Affiliated Hospital of Medical School Nanjing University Nanjing Jiangsu Province China; ^2^ Division of Trauma and Acute Care Surgery, Jinling Hospital, School of Medicine Southeast University Nanjing Jiangsu Province China; ^3^ Institute of Chemicobiology and Functional Materials, School of Chemistry and Chemical Engineering Nanjing University of Science and Technology Nanjing Jiangsu Province China; ^4^ Key Laboratory of Intestinal Injury, Research Institute of General Surgery, Affiliated Jinling Hospital Medical School of Nanjing University Nanjing Jiangsu China

## Abstract

Neutrophil extracellular trap (NET) has been confirmed to be related to gut barrier injury during intestinal ischaemia–reperfusion (II/R). However, the specific molecular regulatory mechanism of NETs in II/R‐induced intestinal barrier damage has yet to be fully elucidated. Here, we reported increased NETs infiltration accompanied by elevated inflammatory cytokines, cellular necroptosis and tight junction disruption in the intestine of human II/R patients. Meanwhile, NETs aggravated Caco‐2 intestinal epithelial cell necroptosis, impairing the monolayer barrier in vitro. Moreover, *Pad4*‐deficient mice were used further to validate the role of NETs in II/R‐induced intestinal injury. In contrast, NET inhibition via *Pad4* deficiency alleviated intestinal inflammation, attenuated cellular necroptosis, improved intestinal permeability, and enhanced tight junction protein expression. Notably, NETs prevented FUN14 domain‐containing 1 (FUNDC1)‐required mitophagy activation in intestinal epithelial cells, and stimulating mitophagy attenuated NET‐associated mitochondrial dysfunction, cellular necroptosis, and intestinal damage. Mechanistically, silencing Toll‐like receptor 4 (TLR4) or receptor‐interacting protein kinase 3 (RIPK3) via shRNA relieved mitophagy limitation, restored mitochondrial function and reduced NET‐induced necroptosis in Caco‐2 cells, whereas this protective effect was reversed by TLR4 or RIPK3 overexpression. The regulation of TLR4/RIPK3/FUNDC1‐required mitophagy by NETs can potentially induce intestinal epithelium necroptosis.

## Graphical abstract

Under intestinal ischaemia–reperfusion, the activated neutrophils infiltrate the intestine and release NETs. NETs initially bind to Toll‐like receptor 4 (TLR4) on the surface of intestinal epithelial cells and further activate the downstream receptor‐interacting protein kinase 3 (RIPK3) protein through TLR4. As a protein kinase, RIPK3 impairs FUN14 domain‐containing 1 (FUNDC1)‐required mitophagy via the phosphorylation of FUNDC1 at Tyr18, which leads to damaged mitochondria in cells that cannot be cleared in time. However, the accumulation of damaged mitochondria results in mitochondrial dysfunction, manifesting as mitochondrial membrane potential loss, ATP synthesis decrease, mt‐ROS overproduction, mPTP opening and Cytc leakage. Irreversible mitochondrial dysfunction triggers the mitochondrial‐dependent necroptosis pathway and ultimately causes intestinal epithelial cellular necroptosis and gut barrier injury.

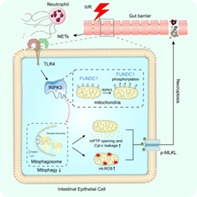

## 1 INTRODUCTION

Intestinal ischaemia–reperfusion (II/R) injury is a serious pathophysiologic process in several clinical conditions, such as mesenteric artery occlusion, abdominal trauma, haemorrhagic shock and intestinal obstruction.^1^ Although restoring blood flow to the ischaemic intestine is essential to prevent irreversible cellular injury, reperfusion may cause a paradoxical increase in intestinal tissue damage in excess of that produced by ischaemia alone.^2^, ^3^ The intestine, a critical organ with a high blood supply, is vulnerable to ischaemia‐ and hypoxia‐induced damage, leading to inflammatory mediator production and damage‐associated molecular pattern (DAMP) release.^4^ The combined effect of inflammatory mediators and DAMPs activates neutrophils, which migrate to the injured site of the intestine, leading to a local inflammatory response and aggravation of tissue injury. Simultaneously, further intestinal barrier disruption results in mucosal shedding and bacterial translocation.^5^ These factors may develop into severe sepsis, septic shock and even multiple organ dysfunction syndrome (MODS).

Neutrophils are an essential element of the innate immune defence against invading microbial pathogens and contribute to acute inflammation and autoimmune pathogenesis. In 2004, a novel mechanism in which neutrophils create extracellular DNA webs, known as neutrophil extracellular traps (NETs), was described for the first time by Brinkmann et al.^6^ NETs are composed mainly of extracellular decondensed chromatin affixed with histones, myeloperoxidase (MPO), and neutrophil elastase (NE) and can capture and destroy infectious microorganisms. However, an uncontrolled or excessive NET formation process may cause cellular damage and adjacent tissue injury.^7^ NETs are a complex of DAMPs that can be recognized by Toll‐like receptors (TLRs).^8^, ^9^ Recently, NETs have been found to take part in the pathogenesis of intestinal, liver and lung injury during I/R diseases.^7^, ^10^, ^11^, ^12^ Strategies targeting NET formation could alleviate ischaemia–reperfusion injury occurring in different organs, as well as inflammatory responses, and improve the survival rate. Our previous studies have demonstrated that NETs may damage the intestinal barrier during II/R injury in a rat model.^13^ However, the biological role and downstream effector pathways of NETs in II/R‐induced intestinal barrier injury are still ambiguous.

Mitochondrial autophagy (mitophagy) is an essential mitochondrial quality control pathway that sequesters impaired or depolarized mitochondria into autophagosomes for lysosomal degradation and maintains mitochondrial homeostasis.^14^, ^15^ Recently, accumulating evidence has demonstrated that disabled mitophagy might serve as a link to II/R injury, where the persistent accumulation of defective mitochondria has been suggested to lead to mitochondrial dysfunction and increased cellular necroptosis.^16^, ^17^, ^18^ Furthermore, several studies revealed that it could be one of the primary causes of the increase in reactive oxygen species (ROS) generation. Excess ROS produced by mitochondria cause the persistent opening of the mitochondrial permeability transition pore (mPTP), leading to mitochondrial membrane potential (MMP) loss, cytochrome‐c (Cytc) leakage, ATP synthesis interruption and eventually initiation of necroptosis.^19^, ^20^, ^21^ Although many lines of evidence have suggested a close association between mitochondrial dysfunction and I/R injury, there is still a strong need to clarify the mechanism and specific upstream pathway of mitochondrial dysfunction, specifically in II/R injury.

Receptor‐interacting protein kinase 3 (RIPK3), a serine/threonine protein kinase, has been demonstrated to be involved in a variety of biological processes, including programmed necrosis, cell cycle regulation, metabolism and immune and inflammatory responses.^22^ Toll‐like receptor 4 (TLR4) could be used as an upstream signalling molecule of RIPK3 activation. Recent studies have demonstrated that RIPK3 drives mitochondrial dysfunction, ER stress and inflammatory factor generation through the TLR4 signalling pathway, independent of necroptosis.^23^, ^24^ At the same time, FUN14 domain‐containing 1 (FUNDC1) appears to play a critical role in mitophagy. FUNDC1 is an integral mitochondrial outer‐membrane protein that interacts with LC3 to increase mitophagosomes and thus maintain appropriate mitochondrial homeostasis in ischaemic and hypoxic conditions.^25^, ^26^ The activity of FUNDC1‐mediated mitophagy is regulated by phosphorylation and dephosphorylation. Previous studies have shown that the upregulation of RIPK3 during cardiac ischaemia/reperfusion injury disrupted ischaemia‐induced mitophagy via the phosphorylation of FUNDC1.^27^ However, the potential interactions between RIPK3 and FUNDC1‐mediated mitophagy in II/R injury have not been reported. In this study, we aimed to investigate the role of NET formation in II/R‐induced intestinal barrier injury. We hypothesized that in II/R, NETs might downregulate mitophagy levels in intestinal epithelial cells via the TLR4/RIPK3/FUNDC1 signalling pathway. Furthermore, as mitophagy is impaired, the excessive accumulation of mitochondrial ROS and Cytc leakage from damaged mitochondria could induce severe mitochondrial dysfunction, ultimately leading to intestinal epithelium necroptosis and gut barrier injury.

## 2 MATERIALS AND METHODS

### 2.1 Ethics statement

This study was carried out in accordance with the Recommendations of Guidelines for Clinical Trials by the Ethics Committee of Jinling Hospital. The study protocol was performed according to the principles of the Declaration of Helsinki and was approved by the Ethics Committee of Jinling Hospital (2022DZGZR‐018). Informed written consent was obtained from all participants before any study‐related procedures. Mice were bred under pathogen‐free conditions in the Research Institute of General Surgery at Jinling Hospital. They were euthanized by carbon dioxide narcosis in accordance with the local and national ethical guidelines on the use of laboratory animals. All animal procedures and experimental protocols were approved by the Institutional Animal Care and Use Committee at Jinling Hospital (2022DZGKJDWLS‐0058).

### 2.2 Patient recruitment

A total of 16 patients with isolated acute superior mesenteric artery occlusion disease were enrolled in this study (Jinling Hospital, Medical School of Nanjing University, Nanjing, China). All patients were treated with surgical resection of the diseased bowel and intestinal ostomy due to ineffective conservative treatment. Specific surgical operations were performed as described in our previous study.^28^ Eight intestinal samples from resection specimens were collected for subsequent analysis. Meanwhile, healthy intestinal tissue from the intraoperatively removed enterostomy of eight patients who underwent definite intestinal anastomosis of ostomy served as controls. Moreover, all serum samples were collected from patients before surgery.

### 2.3 Animal study

*Pad4* ^*fl/fl*^ C57BL/6 mice (#026708) and Mrp8‐Cre (S100A8‐Cre) C57BL/6 mice (#021614) were purchased from the Jackson Laboratory (Bar Harbor, ME) through the Model Animals Research Center of Nanjing University. *Pad4* ^*fl/fl*^ mutant mice were crossed with Mrp8‐Cre mice to generate neutrophil‐specific *Pad4*‐knockout (*Pad4* ^*fl/fl*^; Mrp8‐Cre, *Pad4* ^*ΔPMN*^) mice. The Model Animals Research Center of Nanjing University provided WT C57BL/6 mice. Male mice at 8–10 weeks of age were used in the experiments (five in each group). During the experiment, the mice were housed in a light‐ and temperature‐controlled room and allowed to freely eat and drink.

The II/R model was established as described in previous studies.^29^, ^30^ Briefly, after an overnight fast (12 h), mice were anaesthetised by isoflurane (oxygen delivered at 1 L/min with 3% isoflurane for induction and 1.5% isoflurane for maintenance) and placed on the operation table. After midline laparotomy, II/R injury was established by occluding the superior mesenteric artery with a nontraumatic vascular clamp for 1 h, followed by 24 h of reperfusion. Animals were fully recovered from anaesthesia within 10 min after the completion of the surgery, yet ibuprofen (200 mg/L drinking water) was provided for postoperative analgesia and they were closely monitored for the following 24 h. After reperfusion, animals were euthanized as described above, and the serum and terminal ileum specimens were harvested.

### 2.4 Enzyme‐linked immunosorbent assay

Human D‐lactate (K667‐100, BioVision), human I‐FABP (CSB‐E08024h, CUSABIO, China), human & mouse IL‐1β (P420B, Thermo Fisher, China), human & mouse IL‐6 (M620, Thermo Fisher), human & mouse TNF‐α (AMC3012, Thermo Fisher) and human & mouse MCP‐1 (MA5‐17040, Thermo Fisher) in serum or intestinal tissue were analysed using ELISA commercial kits according to the manufacturer's protocols.

### 2.5 Quantification of NETs

Since NETs contain both CitH3, MPO and DNA, a CitH3‐DNA or MPO‐DNA complex ELISA was used to detect and quantify soluble NETs in human and mouse serum as described in our previous study.^31^ Briefly, 96‐well plates were coated with anti‐CitH3 (ab5103, Abcam) or anti‐MPO (ab272101, Abcam) antibodies overnight at 4°C. The plates were then blocked with 2% BSA at room temperature for 2 h. After washing three times, the sample was added to the wells with incubation buffer containing a peroxidase‐labelled anti‐DNA mAb (Cell Death ELISA^PLUS^, 11774425001, Roche, Switzerland). After incubation for 2 h, the plates were washed three times. Peroxidase substrate (ABTS) was added, and absorbance was measured by a microplate reader (FilterMax 3, Molecular Devices) at 405 nm. Values for soluble NET formation are expressed as the percentage increase in absorbance above the control.

### 2.6 Human neutrophil isolation

Isolation of human neutrophils was performed according to our previous protocols.^32^ EDTA anti‐coagulated peripheral blood (5 mL) collected from healthy volunteers after informed consent was layered on Polymorphprep (Axis‐Shield, Oslo, Norway) and centrifuged for 30 min at 500*g*. The lower layer containing the neutrophils was collected into a new centrifuge tube and washed with phosphate‐buffered saline (PBS). After centrifugation (450 × g, 5 min), red blood cells were lysed with RBC lysis buffer (Solarbio, Beijing, China). Isolated neutrophils were then suspended in RPMI 1640 medium (11875‐093, Gibco) containing 1% HEPES buffer (15630‐080, Gibco). The institutional ethics committee at Jinling Hospital approved the protocol.

### 2.7 Cell culture and intestinal monolayer barrier establishment

Caco‐2 cells were purchased from the Cell Bank of the Chinese Academy of Sciences (Shanghai, China). All cells were cultured and maintained using DMEM (10‐013‐CVR, Corning, China) supplemented with 10% FBS (10099‐141, Gibco, USA). Experiments were performed using cells between passages 30 and 50 to maintain continuity of morphological and phenotypical characteristics.

To establish intestinal monolayer barriers, Caco‐2 cells (1 × 10^5^ per well) were cocultured using 12‐well plates containing 0.4‐μm polyester membrane Transwell inserts (3401, Corning Costar Corporation) with 0.5‐mL culture medium in the upper chamber and 1.5‐mL culture medium in the bottom chamber of the Transwell system. Cells were grown to produce confluent monolayers for 21 days, and the culture medium was replaced daily.

### 2.8 NETs isolation and treatment

Freshly isolated neutrophils were seeded in six‐well culture plates at the concentration of 3 × 10^6^ cells per well and stimulated with PMA (100 nM, P1585, Sigma‐Aldrich). After incubation for 4 h at 37°C in a 5% CO_2_ incubator, each well was carefully washed twice with 1 mL PBS and resuspended in DMEM (17‐207‐CV, Corning). The suspension was then centrifuged at 200*g* for 5 min at 4°C to remove whole cells and debris. Following centrifugation, the supernatant was collected and set aside. NETs isolates were quantified by evaluating the DNA concentrations by the Quant‐iT™ PicoGreen™ dsDNA Assay kit (P11496, Thermo Fisher).^33^, ^34^

The isolated NETs were diluted in DMEM to reach the desired concentration (200 and 400 ng/mL). Caco‐2 cells were incubated with NETs or solvent vehicles for different time points. DNase I (1 mg/mL, 10104159001, Roche, Switzerland) was added before coincubation to degrade NETs in the coculture system. To assess whether NETs activate necroptosis, the necroptosis inhibitor Necrostatin‐1 (Nec‐1, 10 μM, HY‐15760, MCE, China) or the apoptosis inhibitor Z‐VAD‐FMK (Z‐VAD, 10 μM, HY‐16658B, MCE, China) was co‐cultured with caco‐2 cells for 2 h before NETs incubation. To determine how NET regulates mitophagy, the cells were pretreated with mitophagy activator Urolithin A (UA, 5 μM, HY‐100599, MCE, China) or inhibitor Cyclosporin A (CsA, 1 μM, HY‐B0579, MCE, China) for 2 h. To investigate the impact of NETs on mitophagic flux, the Caco‐2 cells were pretreated with Bafilomycin A1 (Baf A1, 10 nM, HY‐100558, MCE, China) for 2 h.

### 2.9 TEER and cell viability

The Caco‐2 cell monolayer integrity was monitored by transepithelial electrical resistance (TEER) using an epithelial voltohmmeter (EVOM2, World Precision Instruments) based on our previous study.^33^ The TEER was calculated according to the formula TEER (Ω · cm^2^) = (Ω cell monolayer − Ω filter [cell‐free]) × filter area. The changes in TEER were calculated with the formula TEER (%) = (TEER/initial TEER) × 100. A cell monolayer with a TEER value over 500 Ω · cm^2^ was used in all experiments.

Cell viability was measured using the Cell Counting Kit‐8 assay (CCK‐8, C0037, Beyotime, China) according to the manufacturer's protocol.

### 2.10 Histology, immunohistochemistry and immunofluorescence

For histology, fresh terminal ileal tissue specimens were fixed in 4% paraformaldehyde and embedded in paraffin. Routine haematoxylin and eosin (H&E) staining and Masson's Trichrome Staining were performed on intestinal paraffin sections. Two independent pathologists examined these sections according to the Chiu scoring system.^35^

For immunohistochemistry, tissues were fixed, embedded and sectioned as described above. After antigen repair, primary antibodies, including anti‐p^Tyr18^‐FUNDC1 (custom‐made from Huabio, China), anti‐TLR4 (ab22048, Abcam) and anti‐RIPK3 (ab62344, Abcam), were added for overnight incubation at 4°C. Following primary and HRP‐conjugated secondary antibody incubation, diaminobenzidine (DAB) was used as a chromogen. Finally, the sections were visualized after counterstaining with haematoxylin. Analysis was done by investigators blind to experimental groups using ImageJ software.

For immunofluorescence, the slides were blocked with 3% BSA for 1 h and incubated overnight at 4°C with the primary antibody. The primary antibodies included anti‐Ly6G (Human: PA5‐84280, Thermo Fisher. Mouse: 65140‐1‐Ig, Proteintech, China), anti‐CitH3 (ab5103, Abcam), anti‐p‐MLKL (AF3904, Affinity, China), anti‐TOM20 (ab186735, Abcam), LC3 A/B (1:1000, 4108S, CST) and anti‐Cytc (ab133504, Abcam). The nuclei were counterstained with DAPI according to the manufacturer's instructions. The cells were photographed by confocal microscope (Nikon Eclipse Ti; Nikon, Japan). Tissue sections were photographed with a fluorescence microscope (IXplore Standard; Olympus, Japan). Two independent investigators performed relative fluorescence intensity analysis with ImageJ software.

### 2.11 Evaluation of intestinal permeability and water content

Intestinal permeability was measured using the fluorescein isothiocyanate (FITC)‐dextran permeability assay. Four hours prior to sacrifice, mice were gavaged 0.5 mL of FITC‐dextran (400 mg/kg, 60842‐46‐8, Sigma). Blood samples were collected by cardiac puncture of anaesthetised mice, and serum was collected after centrifugation. The fluorescence intensity was measured with a multifunctional microplate reader (FilterMax 3, excitation/emission, 485/525 nm). The FD4 concentration in each serum sample was calculated based on the standard curves.

Intestinal water content was measured to determine gut oedema. Briefly, ileal tissue (approximately 5 cm) was obtained after the mice were sacrificed. Tissue weight was measured after drying for 48 h in an oven at 80°C. The intestinal tissue water content (%) was calculated using the formula: %water content = ([wet–dry weight]/wet weight) × 100.

### 2.12 Transmission electron microscopy

Microvilli, epithelial cell junctions, mitochondrial morphology and mitophagosomes of intestinal epithelial cells were observed by TEM (Hitachi H‐600, Japan). The intestinal tissues were fixed using 4% glutaraldehyde and then treated with 2% osmium tetroxide. After dehydration through graded ethanol solutions, the samples were embedded in epoxy resin and sectioned. The thin sections were stained with uranyl acetate and lead citrate for TEM observations.

### 2.13 MMP (Δ*Ψ*m), ATP generation and mitophagy analysis

The MMP was detected by the JC‐1 Assay Kit (C2003S, Beyotime, China) following the manufacturer's instructions. Briefly, cells were incubated with JC‐1 staining solution for 20 min at 37°C and then washed twice with JC‐1 buffer solution. A fluorescence microscope (IXplore Standard; Olympus, Japan) was used to detect and photograph JC‐1 aggregate (excitation and emission wavelengths were 525 and 590 nm, respectively) and JC‐1 monomer (excitation and emission wavelengths were 490 and 530 nm, respectively).

Intracellular ATP generation was detected using an ATP measurement kit (S0027, Beyotime, China). Cells were harvested, lysed and centrifuged, and then the supernatant was collected. Subsequently, an ATP detection working solution (100 μL per well) was added to 96‐well plates, and then 20 μL samples or ATP standards were added for detection by a luminometer (FilterMax 3, Molecular Devices). The ATP concentration for each sample was calculated based on a standard curve.

For mitophagy quantification, the Caco‐2 cells were transfected with mt‐Keima probe^36^, ^37^ (mitochondria‐targeted monomeric Keima‐Red), a pH‐dependent fluorescent protein from Genechem (Shanghai, China). mt‐Keima emits green light (ex 458 nm) at a neutral pH and red light (ex 534 nm) in acidic lysosomes. Then, live cells were stained with Hoechst 33342 (R37165, Thermo Fisher) for nucleus staining and observed under a confocal microscope (Nikon Eclipse Ti; Nikon, Japan). ImageJ software calculated the mitophagy index as the ratio between the red and green areas.

### 2.14 Mitochondrial ROS detection

Mitochondrial ROS (mt‐ROS) and superoxide were measured using MitoSOX Red mitochondrial superoxide indicator (5 μM, M36008, Thermo Fisher). Fluorescence microscopy (Olympus) was used to examine the mt‐ROS concentration at an excitation wavelength of 510 nm and emission at 580 nm.

### 2.15 Gene silencing and overexpression

The shRNAs and overexpression plasmids for *FUNDC1*, *TLR4* and *RIPK3* were constructed and purchased from Genechem (Shanghai, China). Following the manufacturer's protocol, Caco‐2 cell transfection was performed using Lipofectamine 3000 reagent (L3000015, Invitrogen) cell transfection. The target‐specific shRNA sequences were as follows: *FUNDC1* shRNA, 5′‐GCAAACTAGTATCTGCTGTAA‐3′, *TLR4* shRNA, 5′‐CCGCTGGTGTATCTTTGAATA‐3′ and *RIPK3* shRNA, 5′‐GCCGGCTCTGGTGACTAAATT‐3′.

### 2.16 Mitochondrial extraction

To isolate mitochondria and extract mitochondrial proteins, mitochondrial extraction kits (Tissues: C3606, Cells: C3601, Beyotime, China) was used according to the manufacturer's protocol. Briefly, cells or tissues were resuspended in 800 μL of Mitochondria Isolate Reagent, incubated on ice for 15 min and then homogenized using a homogenizer. The homogenate was centrifuged at 4°C for 5 min at 600 × g. The supernatant was then transferred into a fresh tube and centrifuged (11,000 × g, 10 min) to pellet mitochondria. The resulting cytosolic supernatant was used for western blot analysis. The mitochondrial pellet was washed with cold PBS and resuspended in RIPA lysis buffer for western blotting.

### 2.17 Western blot

Total proteins isolated from intestinal tissue or cells were extracted using lysis buffer (SD001, Invent Biotechnologies). Proteins were separated by SDS–PAGE and transferred to polyvinylidene difluoride membranes (ISEQ00010, Merck Millipore, Ireland). The membranes were blocked with 5% skim milk for 2 h and then incubated with the respective primary antibody overnight at 4°C. The following primary antibodies were used: CitH3 (1:2000, ab281584, Abcam), H3 (1:2500, ab1791, Abcam), p‐MLKL (1:1000, ab187091, Abcam), MLKL (1:2000, 66,675‐1‐Ig, Proteintech, China), claudin‐1 (1:1000, ab15098, Abcam), occludin (1:1000, ab216327, Abcam), GAPDH (1:5000, ab181602, Abcam), Ly6G (1:1000, 87048S, CST), p62 (1:1000, 5114S, CST), LC3 A/B (1:1000, 4108S, CST), TOM20 (1:5000, 11,802‐1‐AP, Proteintech, China), TIM23 (1:1000, 11,123‐1‐AP, Proteintech, China), Cytc (1:1000, DF6457, Affinity, China), Parkin (1:1000, ab77924, Abcam), BNIP3 (1:1000, ab10433, Abcam), p^Ser17^‐FUNDC1 (1:1000, PA5‐114576, Thermo), FUNDC1 (1:1000, ab224722, Abcam), TLR4 (1:1000, ab13556, Abcam) and RIPK3 (1:1000, ab62344, Abcam). p^Ser13^‐FUNDC1 (1:500, Abgent, Suzhou, China) and p^Tyr18^‐FUNDC1 (1:500, Huabio, Hangzhou, China) polyclonal antibodies were custom made by immunizing a rabbit with purified FUNDC1 phospho‐peptides followed by affinity purification according to previous study.^27^, ^38^ Next, membranes were incubated with HRP‐conjugated secondary antibody, and protein bands were detected using enhanced chemiluminescence luminous fluid (P10300, NCM Biotech, China). The band intensities were analysed using ImageJ software.

### 2.18 Immunoprecipitation

Immunoprecipitation was performed using a Protein A/G immunoprecipitation kit (P2179M, Beyotime, China). Briefly, cells were lysed with precooled IP buffer for 20 min, and the supernatant was collected after centrifugation. Protein A/G beads were added to every 500 μL supernatant to remove nonspecific binding. After centrifugation, the primary antibody was added and incubated at 4°C overnight. Protein A/G beads were then added and set for another 2 h. The immunoprecipitated complex was collected by centrifugation and washed four times. Samples were boiled after adding loading buffer, and the supernatant was collected after centrifugation and subjected to WB analysis.

### 2.19 Statistical analysis

Statistical analyses were performed using GraphPad Prism version 9.31 (GraphPad Software, Inc., La Jolla, CA). All data are expressed as the mean ± standard deviation (SD) or median with interquartile range. All variables were examined for normal distribution and homogeneity of variance (Shapiro–Wilk test, Levene test). For clinical samples, the *t*‐test was used for data in line with the normal distribution, while the Wilcoxon test examined non‐normally distributed data. Survival curves were created using the Kaplan–Meier method, and the log‐rank test compared differences. For the mouse and cell studies, data are analysed by one or two‐way ANOVA for one or two‐factor experiments or three‐way ANOVA for three factor experiments followed by a post hoc Tukey's test. A *p‐value* less than 0.05 was considered significant (*p < 0.05, **p < 0.01, ***p < 0.001).

## 3 RESULTS

### 3.1 Increased intestinal NET formation and necroptosis activation in II/R patients

**FIGURE 1:**
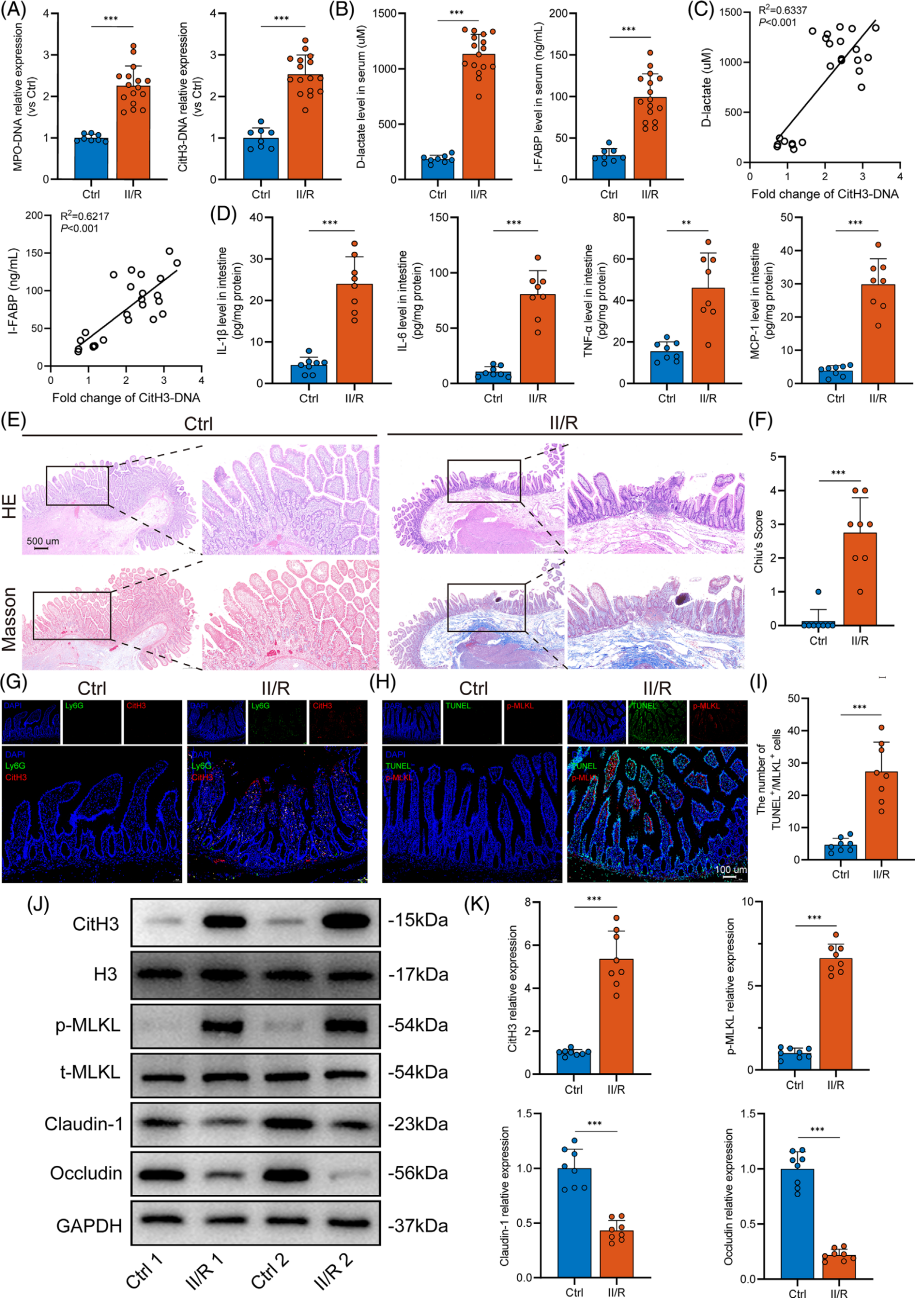
Elevated NET levels are associated with intestinal necroptosis and gut barrier dysfunction in II/R patients. (A) Circulating MPO‐DNA and CitH3‐DNA complexes in healthy controls and II/R patients were analysed by ELISA. (B) Serum D‐lactate and I‐FABP were analysed by ELISA. (C) Correlation between circulating NETs biomarker (CitH3‐DNA) and circulating intestinal injury biomarkers (D‐lactate and I‐FABP). (D) Intestinal inflammatory factors (IL‐1β, IL‐6, TNF‐α and MCP‐1) detected by ELISA. (E,F) Comparisons of the histopathological changes using H&E and Masson staining and the Chiu scoring system. Scale bars = 500 μm. (G) Neutrophil infiltration (Ly6G) and NET formation (CitH3) were assessed by immunofluorescent staining. Scale bars = 100 μm. (H,I) The expression levels of TUNEL and p‐MLKL (a necroptotic biomarker) were analysed through immunofluorescence staining, and the numbers of TUNEL^+^ and p‐MLKL^+^ cells were counted. Scale bars = 100 μm. (J,K) NET formation (CitH3), necroptosis (p‐MLKL, MLKL) and intestinal tight junction proteins (claudin‐1 and occludin) were analysed by western blotting. Grayscale values were measured and quantified using ImageJ software. CitH3, citrullinated histone H3; H&E, haematoxylin–eosin; H3, histone H3; I‐FABP, intestinal fatty‐acid binding protein; II/R, intestinal ischemia–reperfusion; IL‐1β, interleukin 1β; IL‐6, interleukin 6; MCP‐1, monocyte chemoattractant protein 1; MPO, myeloperoxidase; NET, neutrophil extracellular trap; TNF‐α, tumour necrosis factor alpha. Data are shown as the means ± SD. *p < 0.05; **p < 0.01; ***p < 0.001.

Our previous studies have reported NET formation in II/R animal models^13^; hence, we first determined whether NETs also develop in human II/R‐induced intestinal injury. As shown in Figure 1A, serum MPO‐DNA and CitH3‐DNA levels, the specific biomarkers of NETs, were significantly higher in the II/R group than that in the control group. At the same time, the levels of serum D‐lactate and intestinal fatty‐acid binding protein (I‐FABP), circulating markers of intestinal damage, were markedly elevated in the II/R group (Figure 1B). Notably, there were significant correlations between NETs biomarkers and intestinal damage markers in serum (Figure 1C). In addition, the expression levels of the inflammatory factors IL‐1β, IL‐6, TNF‐α and MCP‐1 were detected using ELISA. The inflammatory factor levels in the intestine of II/R patients were increased and significantly higher than those in the control group (Figure 1D), and the differences were statistically significant.

To further determine whether the intestinal injury occurred after II/R, H&E, Masson's trichrome staining and the corresponding Chiu scoring system for intestinal histology were evaluated. The II/R group exhibited worse intestinal morphological injury and an increased Chiu score compared to the controls (Figure 1E,F). Immunofluorescence staining also demonstrated that neutrophil infiltration and NET formation (Ly6G^+^ and CitH3^+^) were significantly elevated (Figure 1G). Furthermore, we evaluated the necroptosis levels in the two groups by immunostaining (Figure 1H,I). The II/R group showed a marked increase in necroptotic cells (the number of TUNEL^+^/p‐MLKL^+^ cells).

Moreover, we investigated the expression levels of intestinal NETs and necroptosis‐associated proteins (Figure 1J,K). Compared to the healthy control group, the protein levels of CitH3, the major component of NETs, were significantly elevated in II/R patients. Increased levels of p‐MLKL, a key protein mediating necroptosis, were also found in II/R patients compared to healthy controls, corroborating the results obtained with immunofluorescence staining. In parallel, tight junction proteins (TJPs), including claudin‐1 and occludin, which are major protein components of the intestinal barrier, were strongly decreased in the II/R group. These results show an increase in intestinal NET formation and necroptosis, and the detrimental role of NETs in intestinal damage during human II/R injury.

### 3.2 NET formation promoted intestinal epithelial cell necroptosis and monolayer barrier dysfunction

**FIGURE 2:**
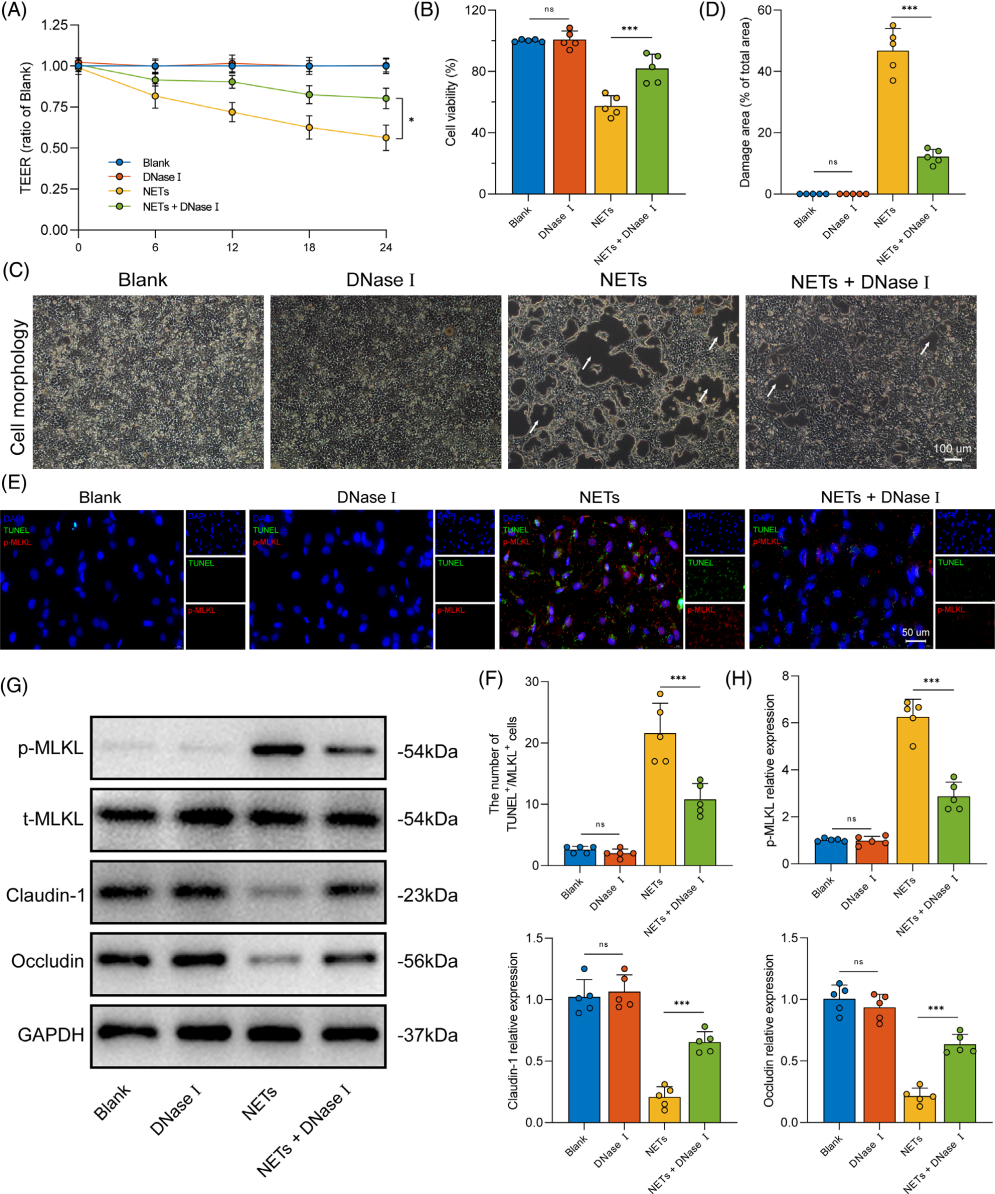
NET formation induced intestinal epithelial cellular necroptosis and monolayer barrier injury in vitro. (A) The barrier function of the Caco‐2 cell monolayer was monitored by TEER over time after NETs (400 ng/mL) treatment. (B) After 24 h co‐incubation with NETs, cell viability was estimated using CCK8 assay, with or without DNase I preincubation. (C,D) The morphology of the Caco‐2 monolayer after NETs (400 ng/mL) treatment with or without co‐stimulation with DNase I, damage area was calculated by ImageJ software, expressed as the percentage of the total area. The damage areas were highlighted with white arrows. Scale bars = 100 μm. (E,F) Necroptosis was assessed by counting the positive TUNEL‐ and p‐MLKL‐stained cells and subjected to statistical analysis. Scale bars = 50 μm. (G,H) Expression levels of necroptosis (p‐MLKL, t‐MLKL) and intestinal tight junction proteins (claudin‐1 and occludin) in Caco‐2 monolayer were examined by western blot analysis. DNase I, deoxyribonuclease I; NET, neutrophil extracellular trap; MLKL, mixed‐lineage kinase domain‐like protein; TEER, transepithelial electrical resistance. Data correspond to the means ± SD, ns, no significance. *p < 0.05; **p < 0.01; ***p < 0.001.

Having identified a clear spatial relationship between NETs and intestinal damage during II/R, we next aimed to investigate the functional relationship between NETs and intestinal epithelium necroptosis using NETs and Caco‐2 co‐culture system. Thus, as indicated, Caco‐2 epithelial barriers and NETs were cocultured and subjected to different time points. The results are shown in Figure 2A,B. TEER and cell viability were markedly decreased by NETs treatment. It should be noted that the most severe injuries were observed at high concentration of NETs (400 ng/mL) treatment for 24 h (Figure S1A,B). Therefore, this concentration and time point were selected for the subsequent experiments.

To validate whether necroptosis is the primary mediator of monolayer barrier damage caused by NETs, we employed necroptosis specific inhibitor Nec‐1 and apoptosis inhibitor Z‐VAD. As shown in Figure S2A–C, Nec‐1 pretreatment significantly reduced the cell morphological damage mediated by NETs and improved cell viability. Z‐VAD partially mitigated NETs‐induced cell morphological injury. Moreover, western blot analysis was conducted to detect the expression levels of necroptosis and TJPs. Upregulation of p‐MLKL and downregulation of TJP levels were observed after the addition of the NETs in Caco‐2 epithelial barriers (Figure S2D,E). In comparison to the Z‐VAD group, pretreatment with Nec‐1 effectively ameliorated claudin‐1 and occludin degradation caused by NETs. These results indicated that the initiation of necroptosis could be the primary factor contributing to the NETs‐induced monolayer barrier damage.

Concurrently, we used deoxyribonuclease I (DNase I), an enzyme used for disintegrating NETs with loss of DNA structures, to pretreat Caco‐2 epithelial barriers before adding NETs. Following DNase I intervention, the cell viability and TEER were significantly improved (Figure 2A,B). Similarly, the administration of DNase I markedly alleviated NETs‐induced cell morphological injury (Figure 2C,D). To examine the number of necroptotic cells, we performed TUNEL and p‐MLKL fluorescence colocalization. Our results show that the number of TUNEL^+^/p‐MLKL^+^ cells in the NETs treatment group increased but significantly decreased in the DNase I preincubation (Figure 2E,F). Moreover, the relative expression levels of necroptosis and intestinal barrier‐associated proteins were detected by western blot analysis (Figure 2G,H; Figure S1C–F). Following the clearance of NETs by DNase I treatment, cell necroptosis (p‐MLKL) levels were significantly decreased. Meanwhile, the expression of TJPs could be preserved. Together, these results confirm that NETs are closely linked to intestinal epithelial cell necroptosis and that clearance of NETs decreases cell necroptosis and attenuates gut barrier injury.

### 3.3 *Pad4* deficiency ameliorates II/R‐induced intestinal epithelial cell necroptosis and barrier dysfunction

The histone‐modifying enzyme, peptidylarginine deiminase 4 (PAD4) is expressed at high levels in neutrophils and plays a critical role in the NET formation. Therefore, neutrophil‐specific *Pad4* (*Pad4* ^*ΔPMN*^) konckout mice explored the potential relationship between NET formation and intestinal epithelium necroptosis. We first profiled the effects of II/R on intestinal epithelial cell necroptosis levels and gut barrier functions at different time points of ischaemia and reperfusion. The results are shown in Figure S3A. A positive correlation was demonstrated between NET formation levels and the time of ischaemia and reperfusion (Figure S3B). Moreover, the 60‐min ischaemia followed by the 24‐h reperfusion group exhibited the highest level of intestinal NET formation and epithelial cell necroptosis (Figure S3B,C). In parallel, this group presented with the most severe intestinal barrier damage, and expressed the lowest TJP levels (Figure S3D,E). Thus, the experimental design mice underwent 60‐min ischaemia followed by 24‐h reperfusion.

**FIGURE 3:**
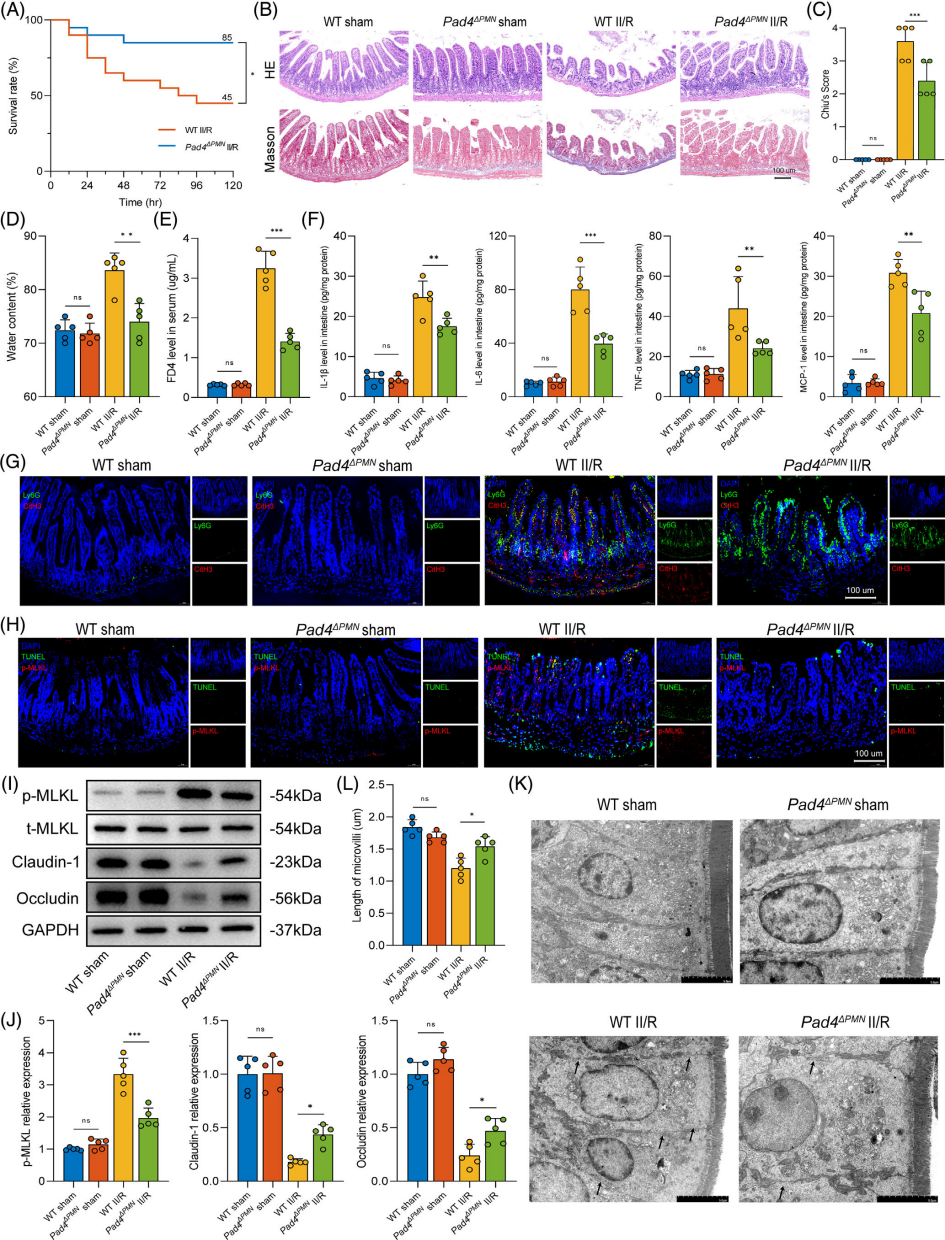
*Pad4* deficiency alleviated II/R‐induced intestinal tissue necroptosis and gut barrier dysfunction. (A) Kaplan–Meier survival analysis after II/R treatment in WT and *Pad4* ^*ΔPMN*^ mice (*n* = 20). The survival rates of the WT and *Pad4* ^*ΔPMN*^ groups at 120 h were 85% and 45%, respectively. (B,C) Representative intestinal pathological images (H&E and Masson staining) and histopathological scores (Chiu's score) after II/R. Scale bars = 100 μm. (D) Intestinal water content was measured to analyse intestinal oedema. (E) Serum FD4 was measured to evaluate intestinal permeability. (F) The expression levels of inflammatory factors (IL‐1β, IL‐6, TNF‐α and MCP‐1) in the intestine detected by ELISA. (G) Immunofluorescent staining for intestinal neutrophil infiltration (Ly6G) and NET formation (CitH3). Scale bars = 100 μm. (H) Necroptosis was assessed by TUNEL and p‐MLKL immunofluorescence staining. Scale bars = 100 μm. (I,J) p‐MLKL, t‐MLKL, claudin‐1 and occludin protein expression was assessed by western blotting. (K,L) Estimate of microvillus length and density changes in intestinal epithelial cells and intercellular tight junctions using transmission electron microscopy. Damaged intercellular tight junctions are shown with arrows. Scale bars = 5 μm. PAD4, peptidylarginine deiminase 4; II/R, intestinal ischaemia–reperfusion; FD4, FITC‐dextran 4. Data are shown as the means ± SD; ns, no significance. *p < 0.05; **p < 0.01; ***p < 0.001.

Survival analysis was performed to evaluate the effect of NETs on the II/R survival rate (Figure 3A). The results showed that the survival rate of *Pad4*‐deficient mice (85%) was significantly improved compared to that of the WT group (45%). To analyse the pathological impairment of the intestine in II/R, intestinal morphological analysis, and Chiu scoring were performed. Histological evaluation by H&E and Masson staining revealed decreased histological damage and Chiu scores in *Pad4* ^*ΔPMN*^ mice that underwent II/R compared with WT II/R mice (Figure 3B,C). Moreover, intestinal permeability, water content and inflammation levels were tested further to evaluate the effect of NETs on gut injury. II/R led to an increased level of intestinal water content (Figure 3D), serum FD4 levels trends (Figure 3E) and inflammatory cytokines (IL‐1β, IL‐6, TNF‐α and MCP‐1; Figure 3F) in the intestine when compared to the sham group. However, decreased generation of these indicators were found in the intestine of *Pad4*‐deficient mice following II/R injury.

Using double immunofluorescence staining, we further evaluated NET formation in terminal ileal tissues after II/R. As shown in Figure 3G, Ly6G‐positive neutrophil infiltration and NET release were elevated in the intestine following ischaemic and reperfusion insult in WT mice. Notably, an increase in Ly6G expression in the *Pad4* ^*ΔPMN*^ II/R group was observed, accompanied by a significant reduction in CitH3 levels. These findings suggest that the neutrophil‐specific deficiency of *Pad4* led to a decrease in NET formation, while neutrophil infiltration remained unaffected.

We next investigated intestinal necroptotic level and gut barrier function in II/R. The necroptotic cells (TUNEL^+^/p‐MLKL^+^ cells) in the intestine were increased after II/R, whereas *Pad4* deficiency significantly reduced the number of necroptotic cells (Figure 3H). Concurrently, western blotting was used to examine the biomarker protein expression necroptosis and TJPs. As shown in Figure 3I,J, II/R led to increased expression levels of p‐MLKL and a highly significant reduction in the expression of TJPs (claudin‐1 and occludin). However, the result was reversed by *Pad4*‐ablation. We explored the cell junctions and microvilli length of intestinal epithelial cells by TEM. According to Figure 3K, the intestinal epithelial cells in the sham group were tightly connected without obvious intercellular space, and the microvilli were neatly organized. However, some damage to intercellular junctional complexes occurred in WT II/R mice, presenting with a widened intercellular space and cell junction defects, accompanied by decreased microvilli length and density (Figure 3L). In the *Pad4* ^*ΔPMN*^ II/R group, cell junction and microvilli injuries were obviously attenuated compared with those in the WT II/R group. These results indicated that NETs drove intestinal epithelial cell necroptosis and gut barrier dysfunction, and the clearance of NETs (*Pad4* deficiency) in the II/R ileum may reduce necroptosis and protection of intestinal barrier function.

### 3.4 Elevated NET formation induced mitophagy suppression and Cytc leakage in the intestine

**FIGURE 4:**
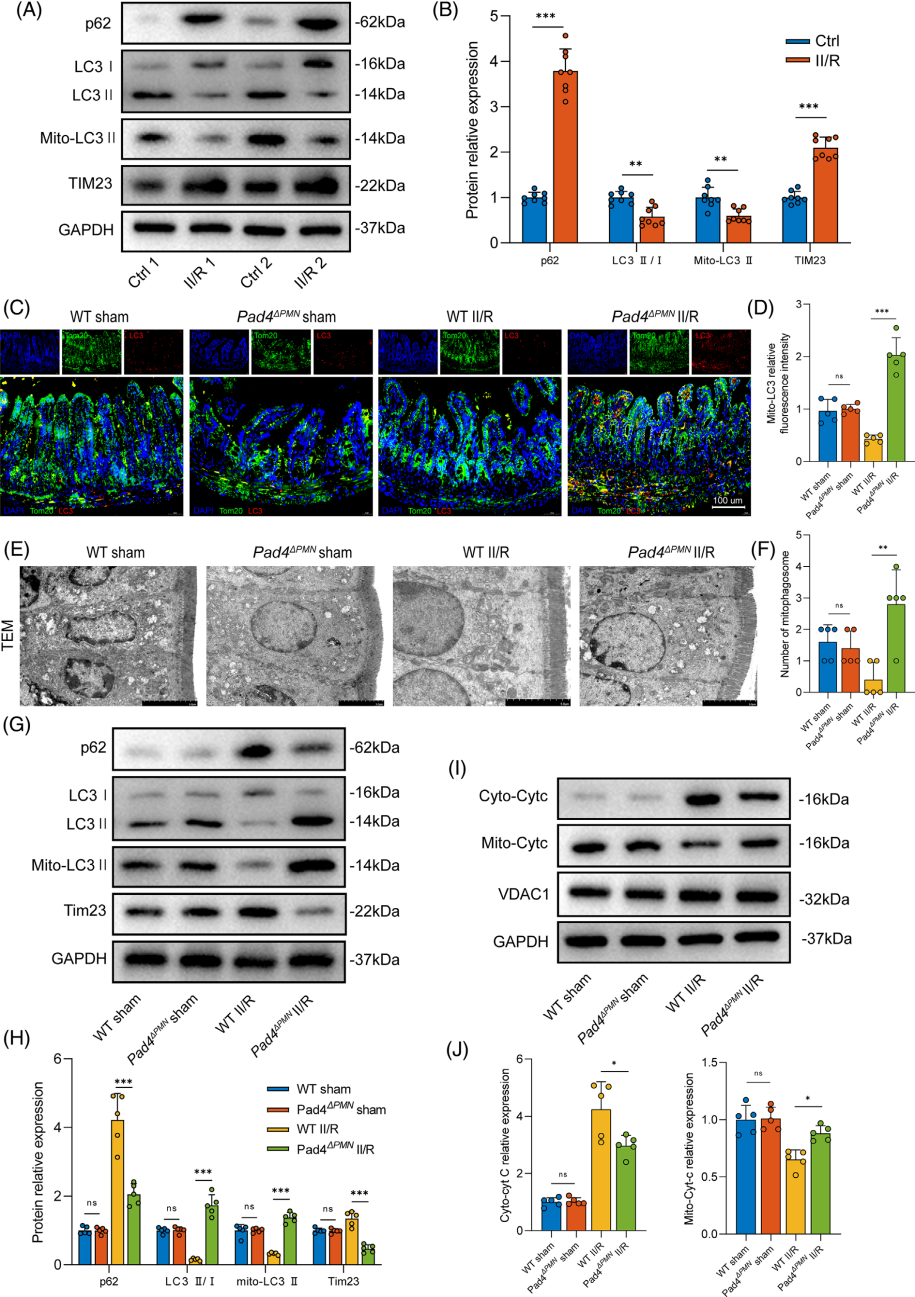
Increased NET levels inhibited mitophagy activation and aggravated Cytc leakage in the intestine. (A,B). Western blotting experiments to assess mitophagy flux (p62, LC3 II/I ratio and Mito‐LC3 II), mitochondrial protein (TIM23) levels in human intestinal tissues. (C,D) The colocalization of LC3 and mitochondria (Tom20) in the intestine in WT and *Pad4* ^*ΔPMN*^ mice. Colocalization events of mitochondria and LC3 puncta are indicated in yellow. Scale bars = 100 μm. (E,F). Qualitative analysis of the number of mitophagosomes in intestinal epithelial cells via TEM. Scale bars = 5 μm. (G,H) Western blots were used to analyse the mitophagy parameters (p62, LC3 II/I ratio and Mito‐LC3 II), mitochondrial biomarkers (Tim23) in animals. (I,J) The Cytc contents in the cytosol and in isolated mitochondria were examined by western blotting. Data are displayed as the mean values with SD. ns, no significance. *p < 0.05; **p < 0.01; ***p < 0.001.

We first assessed the levels of mitophagy‐associated proteins in human intestinal tissue specimens by western blotting (Figure 4A,B). Compared with the control group, p62 levels increased while the mito‐LC3 II decreased in the II/R group. Furthermore, increased levels of mitochondria‐associated protein TIM23 could be observed under II/R injury. To further probe the relationship between NET formation and mitophagy in the intestine, immunofluorescence and TEM were used to observe mitophagy level changes in WT and *Pad4* ablation mice. Our data demonstrated that the fluorescence intensity of mitochondria and LC3 colocalization decreased in WT II/R mice, while these changes were rescued in *Pad4* ^*ΔPMN*^ II/R mice (Figure 4C,D). TEM imaging revealed a reduced number of mitophagosomes in the II/R group compared with the sham group, and *Pad4* deficiency ameliorated the II/R‐induced reduction in mitophagosomes (Figure 4E,F). Subsequently, western blotting was used to detect mitophagy‐associated proteins (Figure 4G,H). The results showed that p62 was significantly increased and the mito‐LC3 II was decreased in response to II/R treatment.

In contrast, *Pad4* deficiency suppressed p62 expression and restored mito‐LC3 II levels. Moreover, there was a noticeable decrease in mitochondrial TIM23 in *Pad4*‐deficient mice, indicating a reduction in associated mitochondria through activation of mitophagy. In addition, we also detected cytosolic and mitochondrial Cytc protein levels (Figure 4I,J). There was a significant elevation in the cyto‐Cytc protein level in WT II/R mice and a substantial moderation of this effect in *Pad4*‐deficient mice. These results confirmed our hypothesis that NET formation inhibits mitophagy and promotes Cytc leakage in the II/R injury setting.

### 3.5 Regulation of mitophagy affects NET‐mediated mitochondrial dysfunction and necroptosis

**FIGURE 5:**
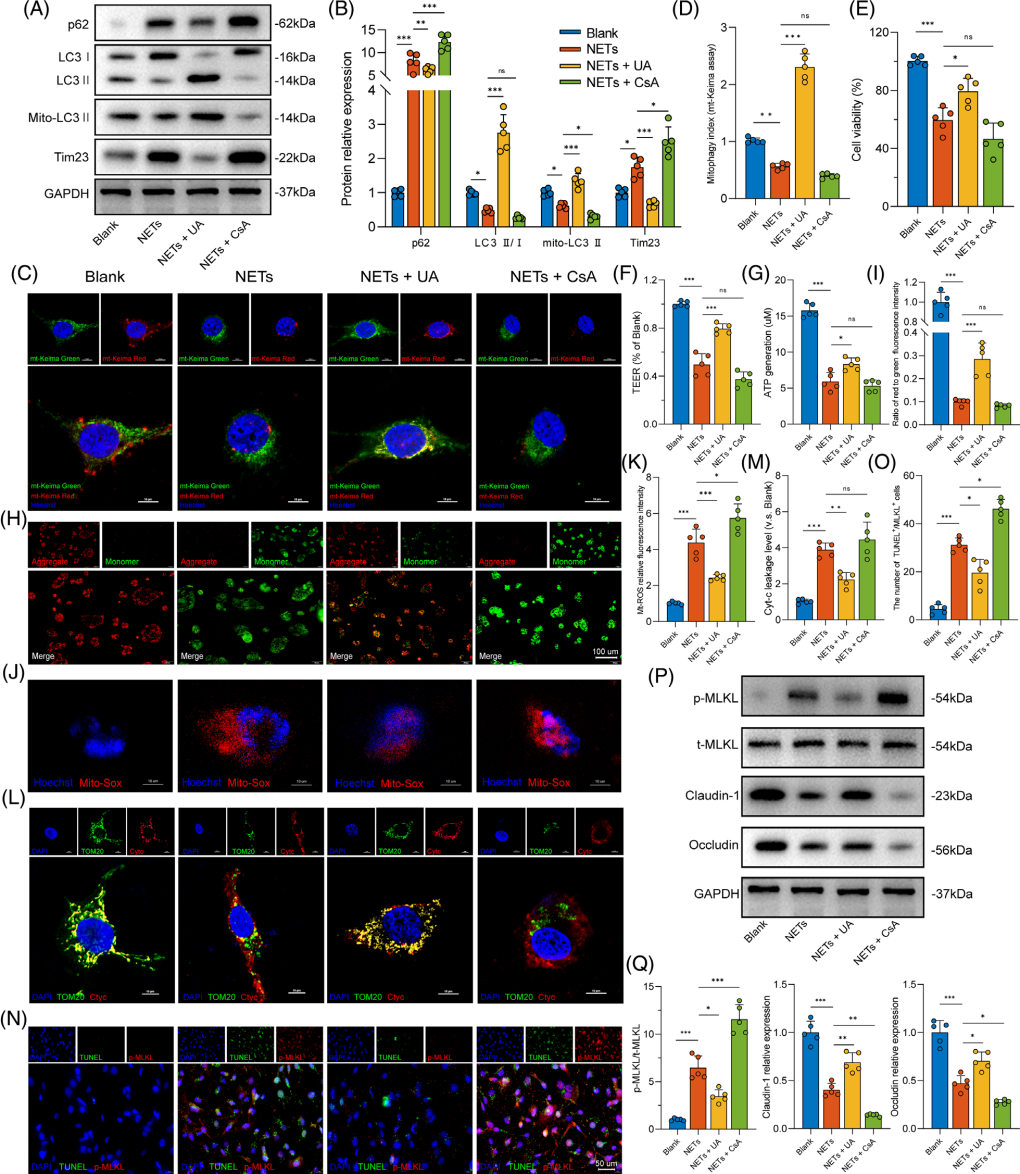
Targeted regulation of mitophagy affected NET‐induced intestinal epithelial cellular mitochondrial dysfunction and necroptosis. (A,B) Western blots were used to analyse the mitophagy parameters after NETs treatment (400 ng/mL) with or without preincubation with UA (5 μM) or CsA (1 μM). (C,D) Mitophagy index was observed using an immunofluorescence assay with the mito‐Keima probe. Fluorescence intensity was analysed by ImageJ software. Scale bars = 10 μm. (E) Cell viability was tested by a cell counting kit‐8 in Caco‐2 monolayers. (F) Intestinal monolayer barrier function was determined by measuring TEER. (G) ATP concentration was detected using an ATP Assay Kit. (H,I) Detection of mitochondrial membrane potential by JC‐1 staining. Red fluorescence indicates healthy mitochondria, whereas green fluorescence indicates collapsed mitochondrial potential. Scale bars = 100 μm. (J,K) Mt‐ROS were measured by MitoSOX Red staining and analysed using ImageJ. Scale bars = 10 μm. (L,M) Cytc leakage was measured through co‐localization analysis of immunofluorescent staining using TOM20 (mitochondria) and Cytc. Images were analysed to quantify the fluorescence intensity using ImageJ. (N,O) TUNEL and p‐MLKL staining were used to label necroptotic Caco‐2 cells after pretreatment with UA or CsA. Scale bars = 50 μm. (P,Q) Western blotting was used to analyse cell necroptosis (p‐MLML/t‐MLKL) and tight junction proteins (claudin‐1 and occludin). CsA, cyclosporin A; ns, no significance; UA, Urolithin A. All the data are expressed as the means ± SD. *p < 0.05; **p < 0.01; ***p < 0.001.

To further investigate the relationship between mitophagy and NET‐induced cell necroptosis, we assessed the effects of mitophagy on mitochondrial function and necroptosis levels in Caco‐2 cells using mitophagy activator Urolithin A (UA) and inhibitor CsA. We first examined the levels of mitophagy in NETs and Caco‐2 co‐culture system with or without UA or CsA. As shown in Figure 5A,B, semi‐quantification of western blots showed that the expression levels of p62 were upregulated. In contrast, LC3 II/I and mito‐LC3 II levels were decreased after NETs with or without CsA treatment. Notably, these results could be reversed after mitophagy activator UA used. Moreover, Baf A1 was used to confirm the changes in mitophagic flux (Figure S4A,B). An elevation in the expression of LC3 II and mito‐LC3 II was observed subsequent to the administration of Baf A1 in both the Blank and NETs + UA groups. Conversely, in the NETs or NETs + CsA group, cells treated with or without Baf A1 exhibited no notable disparities in the LC3 II ratio or mito‐LC3 II level. In addition, mitophagy activity was also detected using an mt‐Keima assay in vitro (Figure 5C,D). The results of relative fluorescence intensity were consistent with the western blotting findings.

We next assessed cellular and mitochondrial function. As shown in Figure 5E,F, cell viability and TEER losses were markedly ameliorated when cells were pretreated with UA. The ATP generation, MMP (Δ*Ψ*m) and mt‐ROS production were detected to further probe mitochondrial function. ATP generation was reduced (Figure 5G), Δ*Ψ*m was destabilized (Figure 5H,I) and mt‐ROS production was upregulated (Figure 5J,K) in cells subjected to NETs treatment. Still, these effects were dramatically reversed by pretreatment with UA. Furthermore, pretreatment of CsA exacerbated mito‐ROS generation and mitochondrial dysfunction. Cytc distribution and leakage were detected through immunofluorescence to evaluate mitochondrial damage. The results indicated that NETs are a strong stimulator that induced the release of Cytc from mitochondria into the cytoplasm (Figure 5L,M). In addition, UA preincubation decreased the expression of Cytc outside the mitochondria, while the use of CsA could aggravate this process, as evidenced by immunofluorescence results.

The Caco‐2 cells were also assayed for necroptosis by immunofluorescence and western blotting. Our data showed that the number of TUNEL^+^/p‐MLKL^+^ cells in the NETs and NETs + CsA groups were increased but were significantly decreased in the UA pretreatment group (Figure 5N,O). Remarkably, p‐MLKL levels were substantially upregulated (Figure 5P,Q), accompanied by the destruction of TJPs claudin‐1 and occludin in the NETs group. However, these changes could be reversed by mitophagy activator UA or be accelerated by mitophagy inhibitor CsA. Taken together, these data indicate that NETs may lead to Caco‐2 cell necroptosis by inhibiting mitophagy levels. Targeted stimulation of mitophagy could decrease NET‐associated intestinal epithelial cell necroptosis.

### 3.6 NETs repress FUNDC1‐required mitophagy via FUNDC1 phosphorylation

The related signalling pathways that NETs employ to inhibit mitophagy during II/R injury are not yet fully understood. To determine which signalling pathway is responsible for NET‐mediated suppression of mitophagy levels, we first examined the expression of classical mitophagy‐related receptors, including Parkin (PINK1‐Parkin dependent mitophagy), FUNDC1 and BNIP3 (receptor‐mediated mitophagy). The data show upregulated expression of BNIP3 in the intestine during II/R (Figure S5A,B). Notably, II/R augmented Fundc1 phosphorylation at Tyr18 and diminished Ser17 phosphorylation. These consequences may contribute to impaired mitophagic activity. However, there was a significant decrease in p^Tyr18^‐FUNDC1 and an increase in p^Ser17^‐FUNDC1 in *Pad4*‐deficient mice during II/R (Figure S5A,B). *Pad4* deficiency resulted in little effect on Parkin and BNIP3 protein expression after II/R injury. In parallel, overall immunohistochemistry findings revealed upregulation of intestinal pTyr18‐FUNDC1 expression during II/R, which was reversed by *Pad4* deficiency (Figure S5C,D). These results indicated that FUDNC1‐required mitophagy could be a target regulated by NET formation.

**FIGURE 6:**
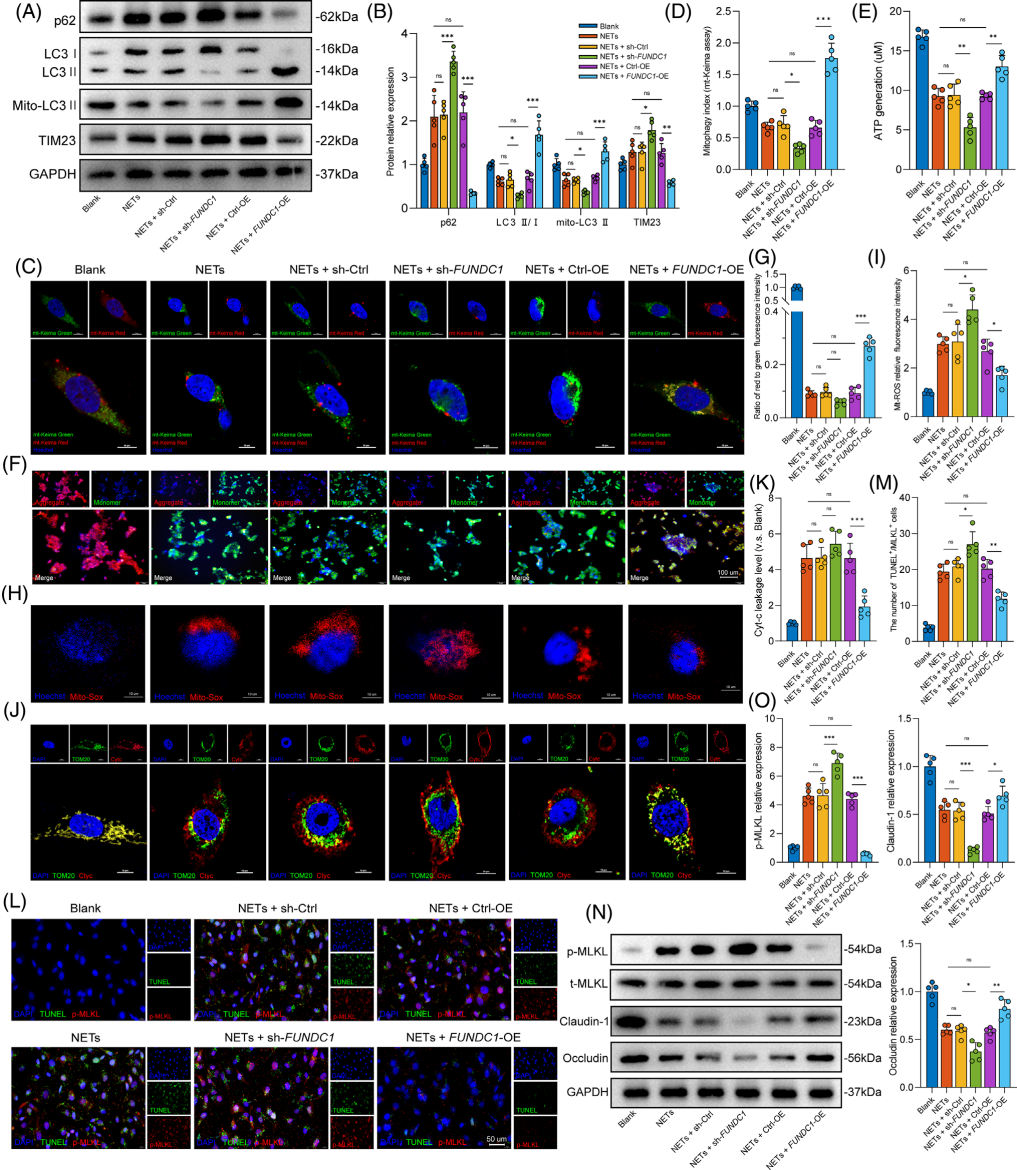
FUNDC1‐required mitophagy is associated with NET‐induced intestinal epithelial cell necroptosis and gut barrier dysfunction. (A,B) Western blotting was used to analyse mitophagy parameters. (C,D) Analysis of mitophagy index in Caco‐2 cells using the mt‐Keima assay. Scale bars = 10 μm. (E) Influence of *FUNDC1* shRNA and overexpression plasmid on ATP generation. (F,G) Changes in mitochondrial membrane potential (Δ*Ψ*m) were detected by JC‐1 fluorescent staining, with red fluorescence indicating JC‐1 aggregates and green indicating JC‐1 monomers. Scale bars = 100 μm. (H,I) Changes in the intensity of red fluorescence emitted by MitoSOX Red in Caco‐2 cells indicated mt‐ROS generation. Scale bars = 10 μm. (J,K) Cytc leakage from mitochondria (TOM20) into the cytoplasm was observed by an immunofluorescence assay. Statistical analysis of Cytc leakage from mitochondria into the cytoplasm. Scale bars = 10 μm. (L,M) Immunofluorescence of p‐MLKL and TUNEL colocalization to identify necroptotic cells. Scale bars = 50 μm. (N,O) The protein levels of necroptosis (p‐MLKL and t‐MLKL) and tight junction protein (claudin‐1 and occludin) in cocultures. Sh, shRNA and OE, overexpression. Data are expressed as the means ± SD. ns, no significance. *p < 0.05; **p < 0.01; ***p < 0.001.

To verify this observation, *FUNDC1* shRNA was used to knockdown FUNDC1 expression. Meanwhile, *FUNDC1*‐overexpressing plasmid was constructed and employed to increase *FUNDC1* expression. We first examined the protein expression levels of FUNDC1 after the transcriptional intervention. As shown in Figure S5E,F, western blot results showed that FUNDC1 shRNA decreased the expression of both phosphorylated FUNDC1 (Ser17 and Tyr18) and total FUNDC1 proteins. Overexpression of FUNDC1 in Caco‐2 cells increased endogenous FUNDC1 expression. Notably, the relative expression of p^Ser17^‐FUNDC1 was significantly reduced in the sh‐FUNDC1 group while little change was observed in the FUNDC1‐OE group. At the same time, the relative expression of p^Tyr18^‐FUNDC1 was elevated in the sh‐FUNDC1 group but decreased in the FUNDC1‐OE group. Next, the levels of mitophagy in Caco‐2 cells were analysed by western blotting and immunofluorescence. WB results showed that *FUNDC1* overexpression reduced p62 and increased the mito‐LC3 II levels, which was concomitant with a decrease in TIM23 expression (Figure 6A,B) at the same time, these results were reversed when *FUNDC1* was knockdown by shRNA. Similarly, the mt‐Keima immunofluorescence results were consistent with the WB findings (Figure 6C,D).

Next, we examined the impact of modulating FUNDC1 protein levels on mitochondrial functional integrity. *FUNDC1* knockdown significantly decreased ATP generation (Figure 6E), lowered the Δ*Ψ*m level (Figure 6F,G), and elevated mt‐ROS production (Figure 6H,I). Moreover, ATP generation was increased, Δ*Ψ*m was stabilized and mt‐ROS overproduction was alleviated in *FUNDC1*‐overexpressing cells. Silencing *FUNDC1* via shRNA increased Cytc translocation from the mitochondria to the cytoplasm, as shown by immunofluorescence (Figure 6J,K) in Caco‐2 cells. In contrast, Cytc leakage was limited by *FUNDC1* overexpression.

We next investigated the effect of FUNDC1 regulation on NET‐induced necroptosis and intestinal monolayer barrier injury during NETs treatment. As the results showed, *FUNDC1* knockdown increased the number of TUNEL^+^/p‐MLKL^+^ cells (Figure 6L,M) and enhanced the expression of p‐MLKL (Figure 6N,O). At the same time, these effects were dramatically reversed by *FUNDC1* overexpression. In addition, *FUNDC1* knockdown resulted in decreased claudin‐1 and occludin expression (Figure 6N,O). Nonetheless, *FUNDC1* overexpression alleviated the loss of TJP protein.

Accordingly, these results indicated that NET‐associated intestinal epithelial cell necroptosis may be mediated through FUNDC1‐required mitophagy, which serves as an endogenous defender of mitochondrial homeostasis and gut barrier function.

### 3.7 The TLR4/RIPK3/FUNDC1 pathway mediates NET‐induced mitophagy suppression and intestinal epithelial cell necroptosis

TLR4 is a critical receptor‐related protein downstream of the NET‐mediated signalling pathway.^39^, ^40^, ^41^ RIPK3 functions as a bridge molecule between NETs and FUNDC1‐required mitophagy. On the one hand, RIPK3 is a major downstream signalling molecule that can be activated by the TLR4 signalling pathway.^23^, ^42^ Meanwhile, as a protein kinase, RIPK3 also impaired FUNDC1‐required mitophagy via the phosphorylation of FUNDC1 at Tyr18.^27^, ^43^ Thus, we speculate that the TLR4/RIPK3/FUNDC1 signalling pathway may be involved in NET‐induced mitophagy inhibition and intestinal epithelial cell necroptosis during II/R injury.

**FIGURE 7:**
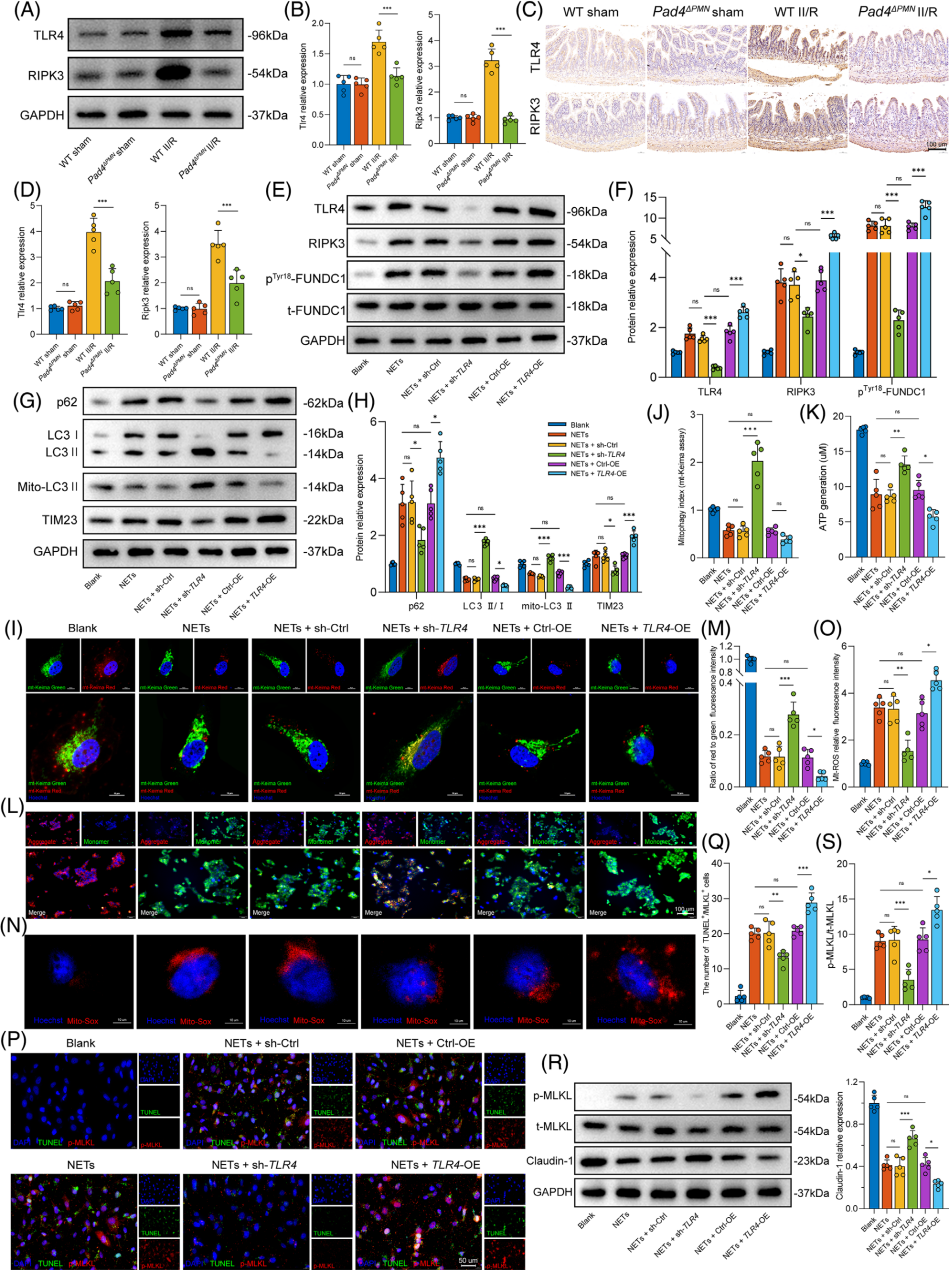
Effects of TLR4 on the protein expression of related pathways and mitochondrial function in intestinal epithelial cells. (A,B) Protein expression of related signalling pathways (TLR4, RIPK3) in WT and *Pad4* ^*ΔPMN*^ mice via western blot. (C,D) Protein expression of TLR4 and RIPK3 in the intestine was detected by immunohistochemistry. The relative expressions were calculated using ImageJ. Scale bars = 100 μm. (E,F) The effects of TLR4 regulation via shRNA or overexpression plasmids on related downstream signal protein expression (TLR4, RIPK3 and p^Tyr18^‐FUNDC1). (G,H) Mitophagy parameters in Caco‐2 cells preincubated with *TLR4* shRNA or overexpression plasmid were detected by western blotting. (I,J) Mitophagic events were observed by fluorescence staining using mt‐Keima assay and examined by confocal microscopy. The relative fluorescence intensity was quantified using ImageJ software. Scale bars = 10 μm. (K) ATP levels were measured by an ATP bioluminescence assay kit. (L,M) JC‐1 staining was performed to detect changes in the Δ*Ψ*m. Scale bars = 100 μm. (N,O) Mitochondrial ROS (mt‐ROS) production was measured by MitoSOX Red staining. Scale bars = 10 μm. (P,Q) The necroptotic cell counts were determined by immunofluorescence, followed by TUNEL and p‐MLKL double staining. Scale bars = 50 μm. (R,S) Necroptotic‐related protein (p‐MLKL) and tight junction protein (claudin‐1) levels in Caco‐2 cells pretreated with *TLR4* shRNA and overexpression plasmids were examined using western blotting. Sh, shRNA and OE, overexpression. Data are presented as the means ± SD. ns, no significance. *p < 0.05; **p < 0.01; ***p < 0.001.

To validate this hypothesis, the expression levels of TLR4 and RIPK3 in the intestine were examined by western blot (Figure 7A,B) and immunohistochemistry (Figure 7C,D) analyses. Significantly higher intestinal TLR4 and RIPK3 expression was detected in WT II/R mice. The upregulation of TLR4 and RIPK3 proteins was substantially reversed in *Pad4*‐deficient mice after II/R injury. These results preliminarily concluded that there certainly is a connection between TLR4, RIPK3 and NET‐induced gut barrier injury.

To further verify the upstream and downstream regulatory relationship of TLR4 and RIPK3, we first transduced Caco‐2 cells with *TLR4*‐specific shRNA or overexpression plasmids and examined the expression of pathway‐associated proteins. As shown in Figure 7E,F, TLR4 protein expression was significantly decreased in Caco‐2 cells following transfection with sh‐*TLR4*, while *TLR4* overexpression markedly increased TLR4 protein expression. Remarkably, both RIPK3 and p^Tyr18^‐FUNDC1 expression were significantly downregulated in Caco‐2 cells transfected with sh‐*TLR4*. Following *TLR4* overexpression plasmid treatment, RIPK3 and p‐FUNDC1 protein expression levels were elevated.

Next, the mitophagy levels of Caco‐2 were evaluated. Immunoblots showed decreased p62 levels, an increased mito‐LC3II and lower TIM23 expression under *TLR4* deletion in Caco‐2 cells (Figure 7G,H), supporting mitophagy activation in the sh‐*TLR4* group. Furthermore, increased mitophagy index (Figure 7I,J) following sh‐*TLR4* treatment suggested an inhibitory effect of TLR4 on mitophagy. Subsequently, mitochondrial function was assessed. Blockade of TLR4 expression via sh‐*TLR4* elevated ATP generation (Figure 7K), reduced Δ*Ψ*m (Figure 7L,M) and alleviated mt‐ROS overproduction (Figure 7N,O). Instead, *TLR4* overexpression reversed these results and caused mitochondrial function impairment.

Moreover, we detected the effect of intervening TLR4 protein expression on cell necroptosis and intestinal monolayer barrier function. The number of TUNEL^+^/p‐MLKL^+^ cells (Figure 7P,Q) was decreased in the sh‐*TLR4* group. Meanwhile, the knockdown of *TLR4* led to reduced expression of p‐MLKL and elevated claudin‐1 protein levels (Figure 7R,S). Conversely, cells overexpressing *TLR4* exhibited higher expression of p‐MLKL and lower claudin‐1 expression after NETs treatment, concomitant with an increased number of TUNEL^+^/p‐MLKL^+^ cells.

**FIGURE 8:**
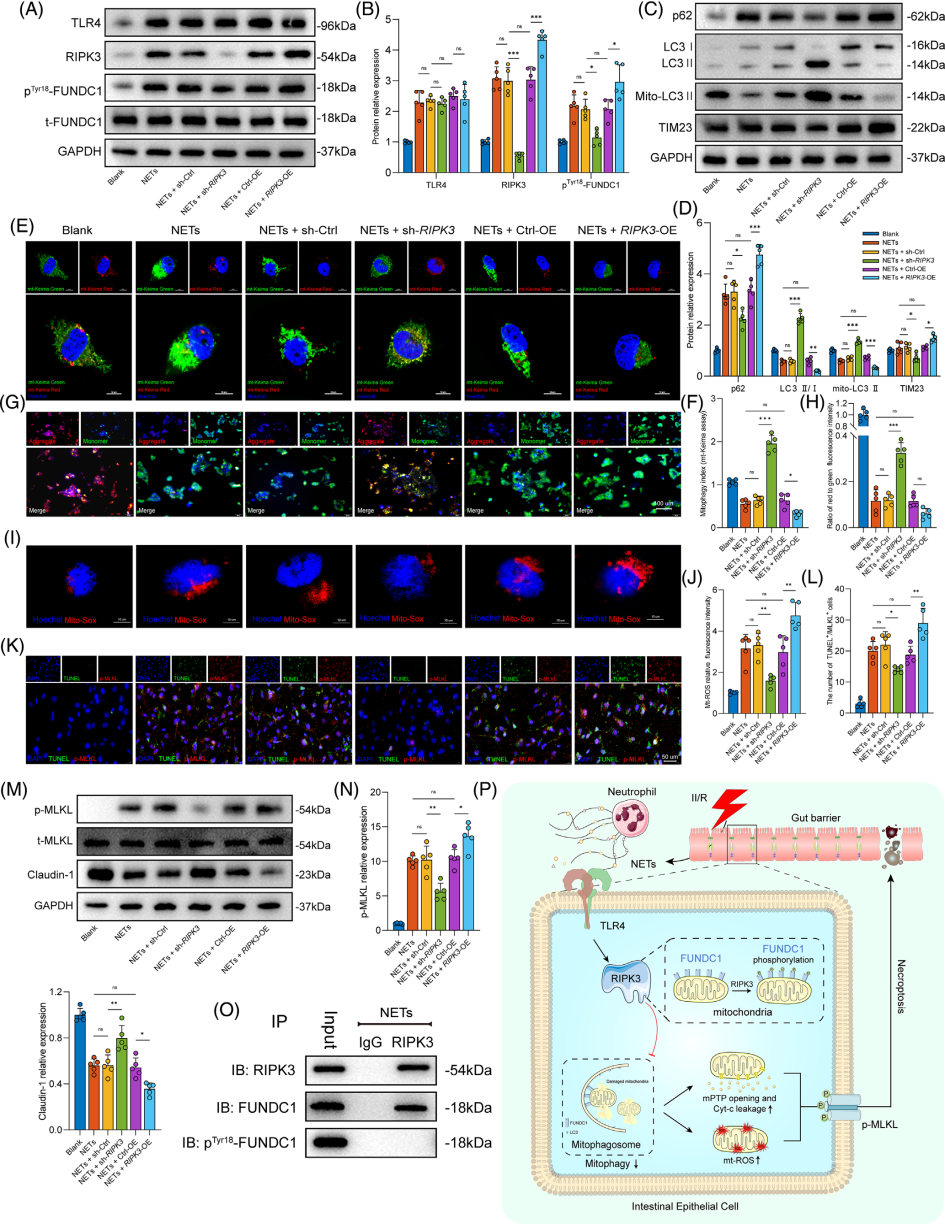
RIPK3 is the downstream protein that plays a key role in regulating NET‐induced mitophagy inhibition and mitochondrial dysfunction. (A,B) Effect of *RIPK3* knockdown or overexpression on the expression of TLR4 and p^Tyr18^‐FUNDC1 via western blot. (C,D) The expression of mitophagy‐related proteins (p62, LC3 and mito‐LC3 II) and mitochondrial protein (TIM23) in *RIPK3* knockdown or overexpression. (E,F) Mitophagy levels were detected by immunofluorescence staining using mt‐Keima probe. Scale bars = 10 μm. (G,H) JC‐1 staining was performed to detect changes in the Δ*Ψ*m. Scale bars = 100 μm. (I,J) Mitochondrial ROS generation was measured using MitoSOX Red staining, and the staining area was analysed using ImageJ. Scale bars = 10 μm. (K,L) Necroptotic cell counts were observed by immunofluorescence staining, followed by TUNEL and p‐MLKL double staining. Scale bars = 50 μm. (M,N) MLKL and claudin‐1 protein expression was analysed by western blot analysis. (O) RIPK3 and FUNDC1 interaction in Caco‐2 cells was assessed by immunoprecipitation (IP) experiments. (P) Schematic diagram showing that NETs aggravated intestinal epithelial necroptosis by regulating TLR4/RIPK3/FUNDC1‐required mitophagy. Sh, shRNA and OE, overexpression. Data correspond to the means ± SD. ns, no significance. *p < 0.05; **p < 0.01; ***p < 0.001.

To explore whether RIPK3 was involved in NET‐induced mitophagy suppression, *RIPK3*‐specific shRNA and overexpression plasmids were constructed and transfected into Caco‐2 cells. After NETs treatment, loss of RIPK3 abated p^Tyr18^‐FUNDC1 expression but did not affect the protein expression of TLR4 (Figure 8A,B). With the overexpression of *RIPK3*, the phosphorylation levels of FUNDC1 were increased again. However, no significant changes in the expression of TLR4 were observed (Figure 8A,B).

Next, mitophagy levels and mitochondrial function were examined. Our data demonstrated that *RIPK3* knockdown significantly increased the levels of mitophagy, as seen with WB (Figure 8C,D) and mt‐Keima immunofluorescence (Figure 8E,F). Moreover, Δ*Ψ*m was sustained (Figure 8G,H), and mt‐ROS overproduction (Figure 8I,J) was ameliorated in *RIPK3*‐silenced cells, but these effects were eventually reversed by *RIPK3* overexpression.

In addition, cell necroptosis and TJPs were evaluated by immunofluorescence and western blotting. Deletion of *RIPK3* resulted in decreased the number of necroptotic cells (Figure 8K,L) and downregulated p‐MLKL expression (Figure 8M,N). In contrast, increased p‐MLKL levels and necroptosis cell counts were observed in the *RIPK3* overexpression group. Moreover, NETs‐induced damage to TJPs was reversed via *RIPK3* deletion (Figure 8M,N), while this protective effect was nullified by *RIPK3* overexpression.

To further explore the correlation between RIPK3 and FUNDC1, the protein interaction in Caco‐2 was detected. The results in Figure 8O show an interaction between FUNDC1 and RIPK3. However, no p^Tyr18^‐FUNDC1 is bound to RIPK3 after NETs treatment. These results suggested that RIPK3 could act directly on unphosphorylated FUNDC1. Moreover, in vitro kinase assay revealed no significant difference in the phosphorylation ability of RIPK3 on FUNDC1 after co‐incubation with NETs (Figure S6A). Taken together, these data indicated that the activation of the TLR4/RIPK3/FUNDC1 pathway could be the mechanism whereby NETs inhibited mitophagy and increased necroptosis in Caco‐2 cells.

## 4 DISCUSSION

In the current study, we first confirmed the increased NET formation and the correlation of NETs with intestinal epithelial cell necroptosis activation and gut barrier dysfunction in intestinal samples from II/R patients. Meanwhile, NETs could elevate Caco‐2 intestinal epithelial cell necroptosis and impair the monolayer barrier in vitro. NET inhibition by *Pad4* deficiency ameliorated II/R‐induced intestinal epithelium necroptosis and barrier dysfunction in vivo, which provided further evidence for a detrimental role of NETs in gut barrier dysfunction during II/R. Another novel finding in the present study was that NETs could prevent FUNDC1‐required mitophagy activation in intestinal epithelial cells. Enhancing mitophagy attenuates NET‐induced mitochondrial dysfunction, cell necroptosis and intestinal damage. Notably, the TLR4/RIPK3/FUNDC1 signalling pathway may participate in NET‐induced mitophagy suppression, mitochondrial dysfunction and cell necroptosis in intestinal epithelial cells (Figure 8P).

II/R injury is an important factor associated with high mortality and prolonged hospitalization. The intestine, a vital organ with a rich blood supply, is highly vulnerable to ischaemia and reperfusion. Intestinal tissues suffering from ischaemia and reperfusion injury can promote the release of ROS and related inflammatory factors, which trigger the accumulation and activation of neutrophils in the intestine.^1^, ^44^ Lingering‐activated neutrophils could further aggravate local intestinal injury, leading to gut barrier damage and translocation of intestinal bacteria and endotoxins.^45^, ^46^ Our previous study in rats demonstrated that II/R injury could cause intestinal inflammation and decrease TJPs.^13^ Similarly, in the present study, we observed increased neutrophil infiltration, increased inflammation and elevated TJP disruption in human II/R patients. In parallel, evidence of gut barrier damage and dysfunction has also been found in animal studies, specifically manifested as exacerbated neutrophil aggregation, increased intestinal permeability, oedema and inflammatory response and disruption of TJPs.

Activated neutrophils participate in the clearance of pathogens via the release of NETs. However, excessive NET formation could result in local tissue damage and serve as a proinflammatory mediator.^47^, ^48^ Circulating NET biomarkers have been reported to be associated with disease severity and clinical prognosis in our previous study.^13^, ^33^ Similar results were also found in the present study, in which circulating NET biomarkers, both MPO‐DNA and the CitH3‐DNA complex, were elevated in serum samples from II/R patients. Moreover, circulating NETs had a positive correlation with biomarkers of intestinal injury (D‐lactate and I‐FABP). Simultaneously, massive neutrophilic infiltration and NET formation were also detected in the intestinal tissue of II/R patients, indicating NET formation's potential role in indicating intestinal injury. To verify the position of NETs in intestinal barrier injury during II/R, we used neutrophil‐specific *Pad4* deficiency mice, which are deficient in NET formation, for further studies. In an II/R animal model, we found that NET inhibition by *Pad4* deficiency increased the survival rate, decreased intestinal inflammatory cytokine levels, alleviated intestinal permeability and relieved intestinal pathological damage. To directly probe the role of NETs in intestinal barrier functions, neutrophils were cocultured with Caco‐2 epithelial barrier cells in vitro. NET formation by neutrophils in concert with Caco‐2 epithelial barrier disruption was observed in the coculture system after NETs stimulation. In addition, NET degradation by DNase I preserved intestinal barrier integrity and function. These results confirmed the detrimental role of NETs in II/R‐induced intestinal barrier dysfunction.

NET formation has been implicated in gut barrier injury, but the precise role has not been elucidated. Dinallo et al. reported that NETs enhanced TNF‐α and IL‐1β production in the intestine by activating the ERK1/2 pathway, which subsequently resulted in mucosal inflammation and intestinal injury.^49^ Our previous study showed that NETs impaired intestinal barrier function during sepsis via the TLR9‐mediated endoplasmic reticulum stress pathway.^33^ In this study, mitophagy suppression was detected in the intestine of II/R patients, and NET formation exhibited a significant negative correlation with mitophagy activation. Moreover, mitophagy is induced to remove damaged mitochondria after ischaemic insult, and activation of mitophagy protects against organ injury by ischaemia–reperfusion.^38^, ^50^, ^51^ Therefore, we speculated that increased NET infiltration inhibits mitophagy activation in intestinal epithelial cells and promotes intestinal injury. Our results showed that *Pad4* deficiency attenuated intestinal barrier dysfunction by activating mitophagy. To further verify the role of mitophagy in NET‐induced intestinal epithelial damage, Caco‐2 cells were preincubated with mitophagy activator (UA) or inhibitor (CsA) before NETs treatment. The results showed that UA pretreatment could decrease necroptosis and alleviate NET‐induced epithelial barrier disruption by antagonizing the inhibitor role of NETs towards mitophagy. On the contrary, these benefits were partially reversed by the mitophagy inhibitor CsA. Therefore, NETs exert crucial roles in mitophagy suppression, which could subsequently lead to intestinal damage and intestinal barrier dysfunction in II/R.

Mitophagy is important in clearing damaged and senescent mitochondria, thereby maintaining mitochondrial quality, cell viability and homeostasis.^20^, ^51^, ^52^ However, impaired mitochondria in cells cannot be removed promptly when mitophagy is inhibited. Excessive accumulation of damaged mitochondria results in mitochondrial dysfunction, which ultimately leads to cell death by necroptosis.^52^, ^53^, ^54^ Some evidence has shown that intestinal epithelial cellular necroptosis is one of the most common causes of gut barrier disruption in II/R.^55^, ^56^ However, it remains unknown whether NETs are involved in mitochondrial dysfunction by inhibiting mitophagy, thus leading to necroptosis of intestinal epithelial cells in II/R. This study observed markedly increased intestinal epithelial cell necroptosis in II/R patients. In an animal model, NET formation had a significant correlation with cellular necroptosis. Furthermore, the necroptotic rate was significantly decreased following *Pad4* deficiency. The results of the in vitro experiment indicated that NET formation suppressed mitophagy levels and aggravated mitochondrial dysfunction, manifesting as MMP loss, mt‐ROS overproduction, decreased mitochondrial ATP synthesis and elevated Cytc leakage. Severe oxidative stress induced mitochondrial dysfunction, ultimately triggering necroptosis, which exhibits similarities to the process of necroptosis activation in myocardial cells under hypoxia/reoxygenation conditions.^57^, ^58^ Furthermore, DNase I or mitophagy activator significantly rescued inhibited mitophagy, alleviated mitochondrial dysfunction and reversed cell necroptosis. While mitophagy inhibitor CsA aggravated mitochondrial dysfunction and further trigger more severe cell necroptosis. Overall, these results provide strong evidence that excessive NETs exacerbate mitochondrial dysfunction by inhibiting mitophagy, resulting in necroptotic cell death.

Mitophagy is differentially regulated depending on disparate stimuli and manifests via specific routes.^51^, ^59^ The depolarized mitochondria are removed by mitophagy through the classic PINK1/Parkin pathway, in which cells are under stress conditions. Under the conditions of hypoxic or mitochondrial membrane potential dissipation, receptor‐mediated mitophagy (such as FUNDC1 and BNIP3) can be activated for the clearance of damaged mitochondria.^25^, ^59^, ^60^ FUNDC1, a highly conserved mitochondrial outer membrane protein, is a mitophagy receptor that regulates mitochondrial dynamics and mitophagy.^25^, ^26^ The role of FUNDC1 phosphorylation at Ser17 contrasts with that of phosphorylation at Ser13 or Tyr18 since the phosphorylation of Ser13 or Tyr18 inhibits FUNDC1‐mediated mitophagy, while phosphorylation at Ser17 increases the interaction between the mitochondria and LC3 and promotes mitophagy.^26^, ^51^, ^61^ In our present study, the expression of related proteins mediating mitophagy was measured. The protein expression levels of FUNDC1, BNIP3 and Parkin in WT mice were detected to varying extents following II/R. However, significant reduction in p^Tyr18^‐FUNDC1 and elevation in p^Ser17^‐FUNDC1 expression were observed in *Pad4*‐deficient mice, while there were no notable differences in the expression of p^Ser13^‐FUNDC1, BNIP3 and Parkin. Thus, we envision that the concerted action of these proteins ultimately manifests as mitophagy suppression in WT II/R mice. Meanwhile, *Pad4* deficiency inhibited NET formation, accounting for FUNDC1 dephosphorylation at Tyr18 and mitophagy upregulation. To confirm this hypothesis, both *FUNDC1* shRNA and overexpression plasmid approaches were used to transfect Caco‐2 cells. Silencing FUNDC1 could decrease mitophagy levels, exacerbate mitochondrial dysfunction, elevate the necroptotic cell rate and enhance intestinal epithelial barrier disruption. Taken together, these data show that FUNDC1‐required mitophagy may play a pivotal role in NET‐induced intestinal epithelial cell necroptosis and gut barrier dysfunction.

The specific molecular mechanism through which NETs regulate FUNDC1‐required mitophagy is complicated and still needs to be completely elucidated. Extracellular histones and DNA, the main components of NETs, can bind TLRs due to their status as critical endogenous DAMPs and thus induce a proinflammatory response and tissue injury.^8^, ^39^, ^62^ As a critical member of the TLR family, TLR4 functions in triggering NET‐induced inflammatory signal transduction.^39^, ^63^, ^64^ Moreover, RIPK3 is an essential downstream key link in the signalling pathway of TLR4, which exerts numerous physiological effects in cell necroptosis modulation, signal transduction mediation and protein activation regulation.^42^, ^65^ More studies have shown the non‐necroptotic functions of RIPK3. Recent studies have found that RIPK3 could drive mitochondrial dysfunction and regulate the inflammatory response through the TLR4 signalling pathway in a necroptotic‐independent manner.^23^, ^42^ As a protein kinase, RIPK3 regulates the reversible protein phosphorylation process in various pathophysiological conditions. Zhou et al. demonstrated that RIPK3 inhibits FUNDC1‐dependent mitophagy by phosphorylating FUNDC1, which leads to mitochondria‐mediated apoptosis in cardiac ischaemia–reperfusion.^27^ Thus, we hypothesized that NETs suppress FUNDC1‐required mitophagy through the TLR4/RIPK3/FUNDC1 pathway. Our current study revealed that the protein expression of TLR4 and Ripk3 was significantly upregulated in WT II/R mice, while their expression exhibited a downward trend following *Pad4* deficiency. To further verify this hypothesis, we constructed shRNA and synthesized an overexpression plasmid to knockdown and overexpress, respectively, *TLR4* and *RIPK3*. This study showed that *TLR4* knockdown decreased both RIPK3 and p^Tyr18^‐FUNDC1 protein expression levels, relieved mitophagy inhibition, ameliorated mitochondrial dysfunction and decreased necroptotic cell levels, while these effects were reversed by *TLR4* overexpression. Furthermore, sh‐*RIPK3* significantly increased the FUNDC1‐required mitophagy activity, whereas upregulated RIPK3 markedly suppressed FUNDC1‐required mitophagy. Notably, changes in RIPK3 expression did not seem to influence TLR4 expression. In addition, immune coprecipitation and the kinase assay of Ripk3 analyses further confirmed the interaction between RIPK3 and FUNDC1. According to the current results, there is an upstream and downstream relationship between the TLR4 and RIPK3 signalling pathways. NETs may suppress FUNDC1‐required mitophagy by regulating the TLR4/RIPK3/FUNDC1 pathway in intestinal epithelial cells during II/R.

This study was subjected to the following limitations. Our study revealed the NET/TLR4/RIPK3/FUNDC1 signalling pathway acting as an essential mechanism that facilitated intestinal epithelial cell necroptosis to induce gut barrier dysfunction, but any other mechanisms cannot be excluded. In addition, the present study investigated the potential role of NETs in the development and progression of intestinal injury during II/R. However, further prospective studies or larger cohorts are warranted to validate our results.

Our study revealed that NETs were involved in II/R‐induced intestinal dysfunction and barrier injury. Elevated NET infiltration was associated with mitophagy inhibition, mitochondrial dysfunction and intestinal epithelial cell necroptosis in II/R patients. NET restriction by *Pad4* deletion ameliorated II/R‐induced mitochondrial dysfunction, cellular necroptosis and intestinal barrier injury by relieving the limitation of FUNDC1‐required mitophagy activation. Moreover, suppressing the NET/TLR4/RIPK3/FUNDC1 signalling pathway may protect against II/R‐induced intestinal barrier damage. Therefore, this study may shed light on the protection of the intestinal barrier in II/R patients.

## AUTHOR CONTRIBUTIONS

**Chengnan Chu**: Data curation; conceptualisation; software; validation; investigation; visualization; methodology; writing—original draft. **Xinyu Wang**: Formal analysis; conceptualisation; resources; data curation; investigation; writing—original draft. **Fang Chen**: Formal analysis; data curation; visualisation; investigation; writing—original draft. **Chao Yang**: Resources; data curation; investigation. **Lin Shi**: Validation; investigation; methodology. **Weiqi Xu**: Software; formal analysis; visualisation; methodology. **Kai Wang**: Data curation; investigation; methodology. **Baochen Liu**: Software; formal analysis; **Chenyang Wang**: Formal analysis; investigation. **Dongping Sun**: Project administration; funding acquisition; writing—review and editing. **Jieshou Li**: Conceptualization; formal analysis; supervision; funding acquisition; visualisation; project administration; writing—review and editing. **Weiwei Ding**: Conceptualisation; formal analysis; supervision; funding acquisition; visualisation; project administration; writing—review and editing.

## FUNDING INFORMATION

This work was supported by the National Natural Science Foundation of China (81770532, 82270587 and 51873087), the Open Project of the State Key Laboratory of Trauma, Burn and Combined Injury, Third Military Medical University (SKLKF202103) and the Innovation Platform of Clinical Medical Center of Jiangsu Province (grant number YXZXA2016006).

## CONFLICT OF INTEREST STATEMENT

The authors declare no conflicts of interest.

## Supporting information


**Data S1:** Supporting Information.

## Data Availability

Data for this study are available from the corresponding author on reasonable request.
