# Supplementary material for: Neutrophil extracellular traps aggravate intestinal epithelial necroptosis in ischaemia–reperfusion by regulating TLR4/RIPK3/FUNDC1‐required mitophagy
Source: Cell Prolif. 2023 Sep 10;57(1):e13538. doi: 10.1111/cpr.13538 (PMC10771116; doi:10.1111/cpr.13538)
Supplement: Supplementary file 1 — Data S1: Supporting Information. [file CPR-57-e13538-s001.pdf]

Supplementary materials

Materials and methods

In vitro RIPK3 kinase assay

To perform in vitro kinase assays for RIPK3, the RIPK3 protein was isolated from cell lysate through immunoprecipitation and subsequently evaluated for the kinase activity of RIPK3 on FUNDC1 phosphoryation utilizing the ADP-Glo^{TM} Kinase Assay Kit (V6930, Promega, USA) following the manufacturer's protocol [1,2]. Briefly, Caco-2 cells were subjected to a cold PBS wash, followed by the addition of 0.5 mL of cell lysis buffer (9803, CST, USA) and incubation on ice for 5 minutes. The resulting lysed cells were transferred to a centrifuge tube and centrifuged at 4 °C, 14,000 g for 10 minutes. The supernatant was collected as the cell lysate. Subsequently, the lysate was treated with anti RIPK3 antibody (ab226297, Abcam, USA) and incubated overnight at 4 °C. Protein A agarose beads (20 μL, P2017, Beyotime, China) was then added and incubated for an additional 2 hours. After centrifugation, the immunoprecipitates were washed thrice with lysis buffer and another 3 times with kinase buffer (9802, CST, USA). The 50 ng immuneprecipitate was resuspended in kinase buffer to a volume of 25 μL. Subsequently, 100 μM ATP and recombinant FUNDC1 (1 μg, ab165435, Abcam, USA) were added as substrate and incubated at a temperature of 37 °C for a duration of 30 minutes. Following the ADP-Glo ™ Kinase Assay manufacturer’s protocol, 25 μL of ADP Glo ™ Reagent was added to the reaction system and incubated at room temperature for 40 minutes to terminate the kinase reaction and deplete any remaining ATP. Subsequently, 50 μL of Kinase Detection Reagent was added and incubated at

room temperature for an additional 30 minutes. The luminescence intensity was then measured using a luminometer (FilterMax 3, Molecular Devices, USA). Using recombinant RIPK3 (ab125566, Abcam, USA) as the control group, the kinase activity of the isolated RIPK3 was determined by calculating the luminescence intensity for each group.

References

1. Chen G, Han Z, Feng D, et al. A regulatory signaling loop comprising the PGAM5 phosphatase and CK2 controls receptor-mediated mitophagy. *Mol Cell.* 2014;54(3):362-377.
2. Chen SM, Zhao CK, Yao LC, et al. Aiphanol, a multi-targeting stilbenolignan, potently suppresses mouse lymphangiogenesis and lymphatic metastasis. *Acta Pharmacol Sin.* 2023;44(1):189-200.

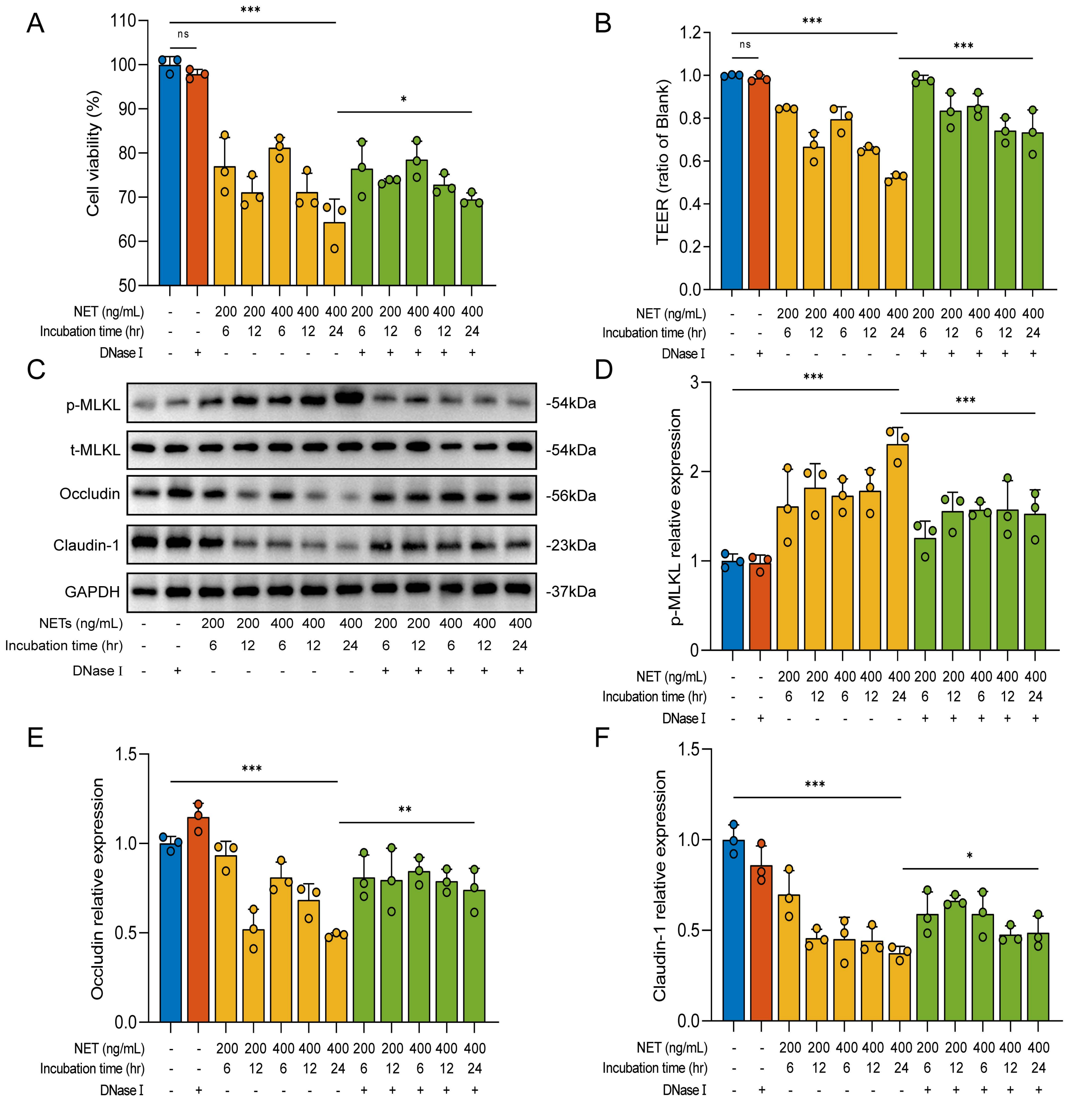
A
Cell viability (%)
B
TEER (ratio of Blank)
C
p-MLKL
t-MLKL
Occludin
Claudin-1
GAPDH
-54kDa
-54kDa
-56kDa
-23kDa
-37kDa
NETs (ng/mL)
Incubation time (hr)
DNase I
D
p-MLKL relative expression
E
Occludin relative expression
F
Claudin-1 relative expression
NET (ng/mL) - - 200 200 400 400 400 200 200 400 400 400
Incubation time (hr) - - 6 12 6 12 24 6 12 6 12 24
DNase I - + - - - - - + + + + +
ns
***
**
*

Supplementary Figure 1

A. Cell viability following NETs treatment was measured by a cell counting kit-8. **B.** TEER values of Caco-2 cells challenged with various concentrations and treatment times of NETs, with or without DNase I treatment. **C-F.** The expression of necroptosis (p-MLKL and t-MLKL) and tight junction proteins (claudin-1 and occludin) were detected via WB analysis. Data are expressed as the means ± SD, ns, no significance, $*p < 0.05$, $**p < 0.01$, $***p < 0.001$.

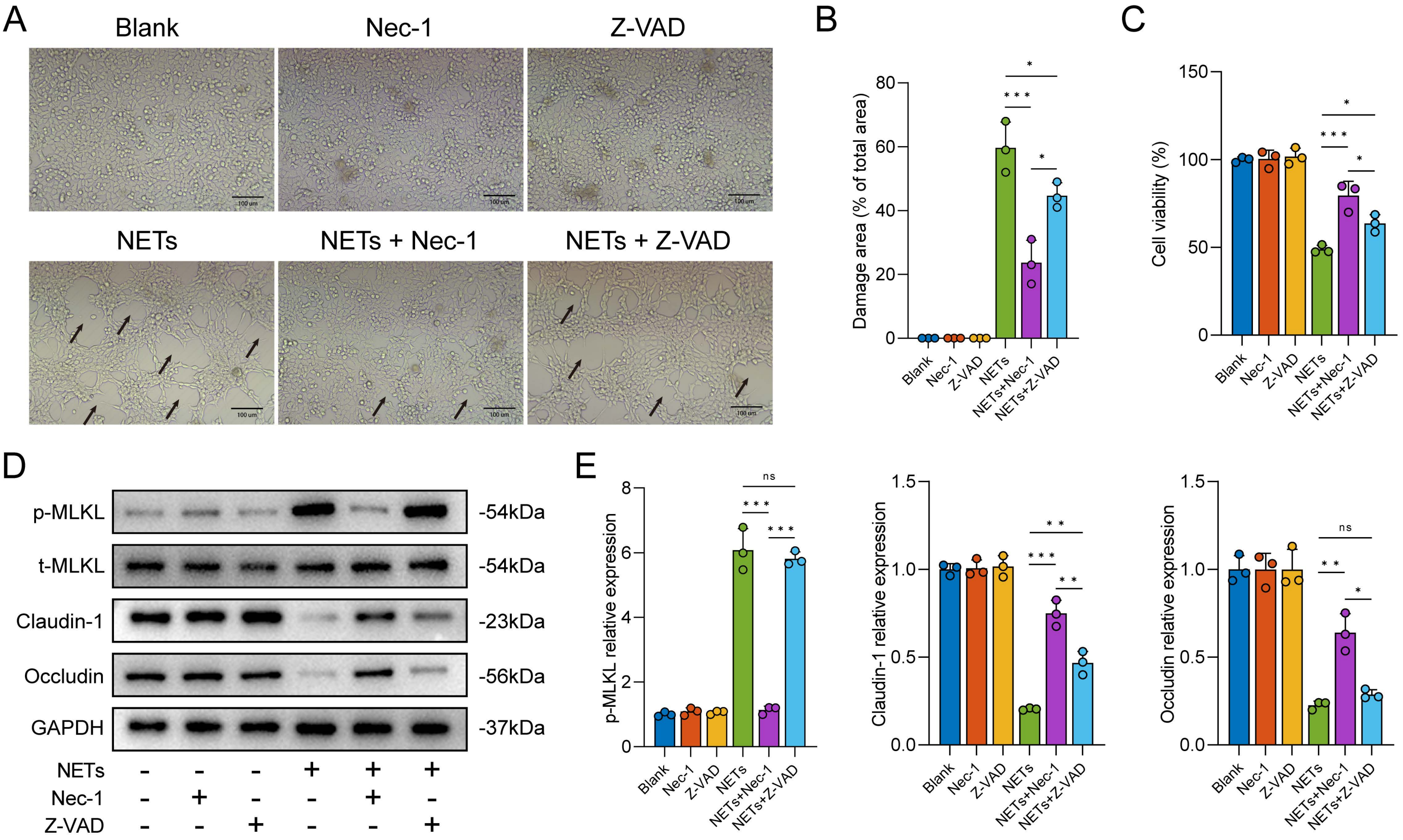

A
Blank
Nec-1
Z-VAD
NETs
NETs + Nec-1
NETs + Z-VAD
100 um
B
Damage area (% of total area)
0
20
40
60
80
Blank
Nec-1
Z-VAD
NETs
NETs+Nec-1
NETs+Z-VAD
C
Cell viability (%)
0
50
100
150
D
p-MLKL -54kDa
t-MLKL -54kDa
Claudin-1 -23kDa
Occludin -56kDa
GAPDH -37kDa
NETs - - - + + +
Nec-1 - + - - + -
Z-VAD - - + - - +
E
p-MLKL relative expression
Claudin-1 relative expression
Occludin relative expression
0.0
0.5
1.0
1.5
ns

Supplementary Figure 2

A-B. The morphological changes of the Caco-2 monolayer were analyzed using ImageJ software to calculate the area of damage after treatment with NETs, with or without co-stimulation with Nec-1 or Z-VAD. Scale bars were set to 100 μm. **C.** Cell viability was estimated using CCK8 assay. **D-E.** The expression levels of necroptosis markers (p-MLKL, t-MLKL) and intestinal tight junction proteins (claudin-1 and occludin) in the Caco-2 monolayer were examined using western blot analysis. Nec-1, Necrostatin-1; Z-VAD, Z-VAD-FMK. Data correspond to the means ± SD, ns, no significance, $*p < 0.05$, $**p < 0.01$, $***p < 0.001$.

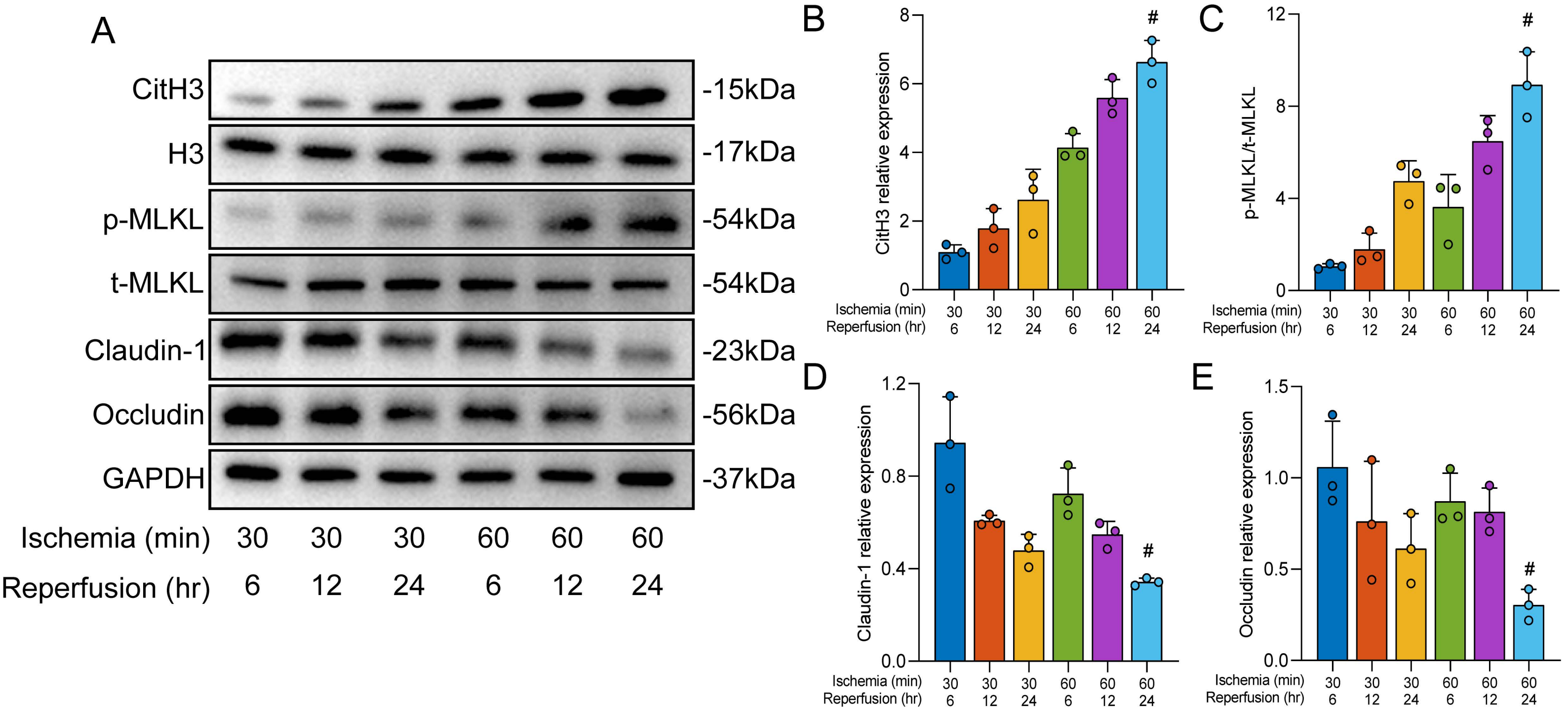
A
CitH3
H3
p-MLKL
t-MLKL
Claudin-1
Occludin
GAPDH
-15kDa
-17kDa
-54kDa
-54kDa
-23kDa
-56kDa
-37kDa
Ischemia (min) 30 30 30 60 60 60
Reperfusion (hr) 6 12 24 6 12 24
B
CitH3 relative expression
C
p-MLKL/t-MLKL
D
Claudin-1 relative expression
E
Occludin relative expression
#

Supplementary Figure 3

A-E. Western blotting was used to assess the effect of different ischemia and reperfusion timepoints on the expression of NET formation (CitH3), necroptosis (p-MLKL/t-MLKL) and tight junction proteins (claudin-1 and occludin). Data are expressed as the means ± SD, # $p < 0.05$ compared with all other groups.

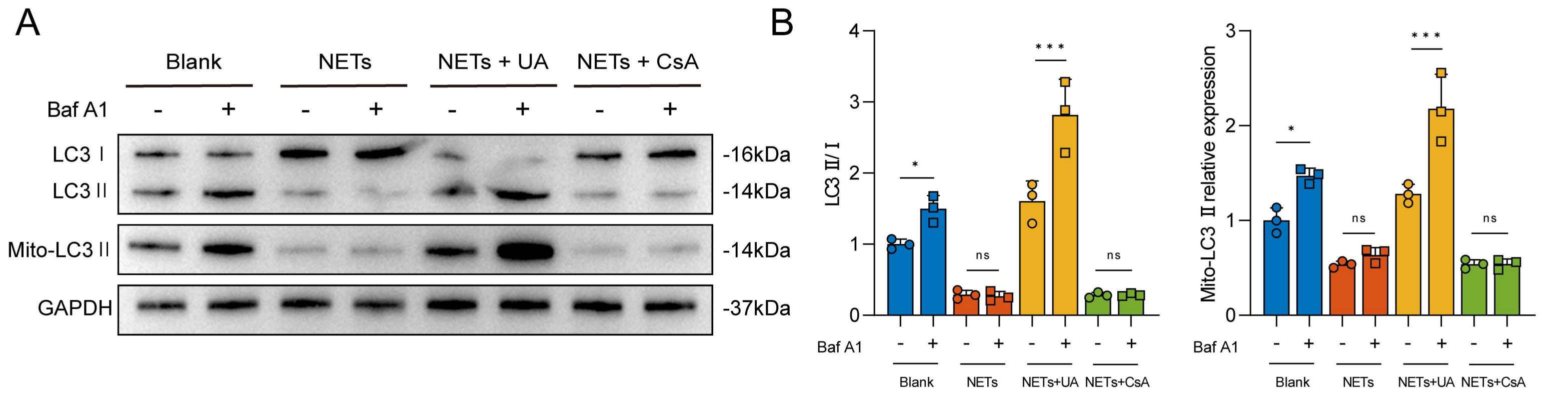
A
Blank
NETs
NETs + UA
NETs + CsA
Baf A1
-
+
LC3 I
LC3 II
Mito-LC3 II
GAPDH
-16kDa
-14kDa
-14kDa
-37kDa
B
LC3 II/ I
Mito-LC3 II relative expression
*
***
ns
Baf A1
Blank
NETs
NETs+UA
NETs+CsA

Supplementary Figure 4

A-B. Cellular mitophagic flux changes were examined by western blot analysis after treatment with Baf A1. The grayscale values of the bands were analyzed using ImageJ. Baf A1, Bafilomycin A1. Data are presented as the means ± SD, ns, no significance, *p < 0.05, ***p < 0.001.

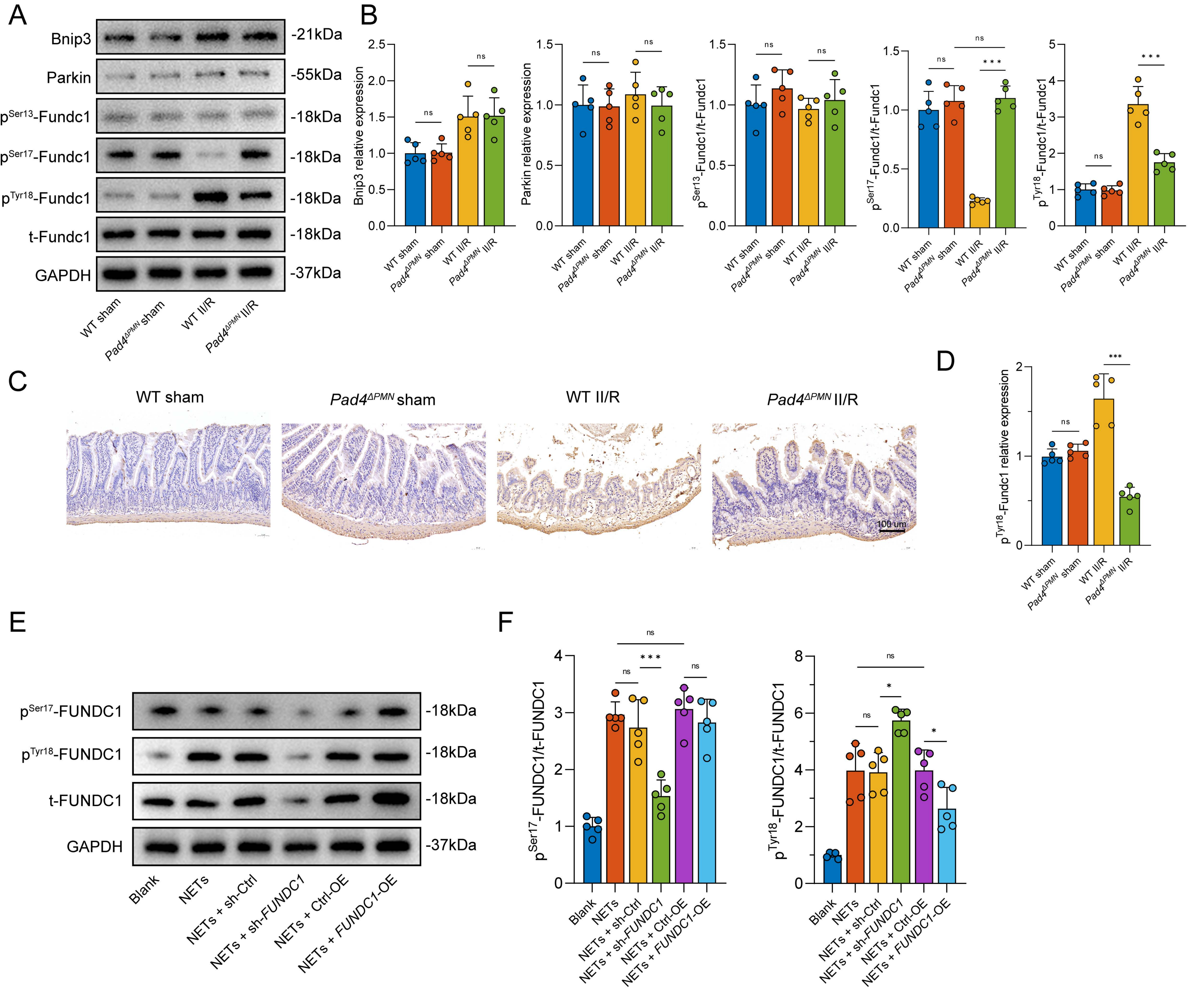
A
Bnip3
Parkin
pSer13-Fundc1
pSer17-Fundc1
pTyr18-Fundc1
t-Fundc1
GAPDH
-21kDa
-55kDa
-18kDa
-18kDa
-18kDa
-18kDa
-37kDa
WT sham
Pad4ΔPMN sham
WT II/R
Pad4ΔPMN II/R
B
Bnip3 relative expression
Parkin relative expression
pSer13-Fundc1/t-Fundc1
pSer17-Fundc1/t-Fundc1
pTyr18-Fundc1/t-Fundc1
ns
***
C
WT sham
Pad4ΔPMN sham
WT II/R
Pad4ΔPMN II/R
100 um
D
pTyr18-Fundc1 relative expression
E
pSer17-FUNDC1
pTyr18-FUNDC1
t-FUNDC1
GAPDH
-18kDa
-18kDa
-18kDa
-37kDa
Blank
NETs
NETs + sh-Ctrl
NETs + sh-FUNDC1
NETs + Ctrl-OE
NETs + FUNDC1-OE
F
pSer17-FUNDC1/t-FUNDC1
pTyr18-FUNDC1/t-FUNDC1
*

Supplementary Figure 5

A-B. Relevant proteins expression of mitophagy receptors (BNIP3, Parkin and Fundc1) were determined through western blotting in WT and *Pad4*$^{\Delta PMN}$ mice. **C-D.** Immunohistochemical staining detected p^{Tyr18}-Fundc1 expression in the intestine. The relative expressions were calculated using ImageJ. Scale bars = 100 μm. **E-F.** The protein expression of FUNDC1 in Caco-2 cells was analyzed after incubation with NETs, either with or without pretreatment using *FUNDC1* sh-RNA or overexpression plasmid. Data are expressed as the means ± SD, ns, no significance, *$p < 0.05$, ***$p < 0.001$.

A

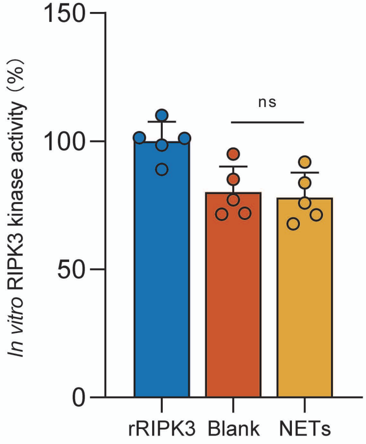

150
100
50
0
In vitro RIPK3 kinase activity（%）
ns
rRIPK3
Blank
NETs

Supplementary Figure 6

A. An in vitro kinase assay of RIPK3 on FUNDC1 phosphoryation. The RIPK3 protein was isolated from cell lysate through immunoprecipitation and evaluated for the kinase activity using the ADP-Glo^{TM} Kinase Assay Kit. Data correspond to the means ± SD, ns, no significance.
